# Clinical Significance and Prognostic Value of Human Soluble Resistance-Related Calcium-Binding Protein: A Pan-Cancer Analysis

**DOI:** 10.3389/fmed.2021.752619

**Published:** 2021-11-16

**Authors:** Jinguo Zhang, Jian Chen, Benjie Shan, Lin Lin, Jie Dong, Qingqing Sun, Qiong Zhou, Xinghua Han

**Affiliations:** Department of Medical Oncology, The First Affiliated Hospital of USTC, Division of Life Science and Medicine, University of Science and Technology of China, Hefei, China

**Keywords:** pan-cancer, sorcin, prognosis (carcinoma), MSI, TMB

## Abstract

The soluble resistance-related calcium-binding protein (sorcin, SRI) serves as the calcium-binding protein for the regulation of calcium homeostasis and multidrug resistance. Although the mounting evidence suggests a crucial role of SRI in the chemotherapeutic resistance of certain types of tumors, insights into pan-cancer analysis of SRI are unavailable. Therefore, this study aimed to probe the multifaceted properties of SRI across the 33 cancer types. The SRI expression was analyzed *via* The Cancer Genome Atlas (TCGA) and Genotype Tissue-Expression (GTEX) database. The SRI genomic alterations and drug sensitivity analysis were performed based on the cBioPortal and the CellMiner database. Furthermore, the correlations among the SRI expression and survival outcomes, clinical features, stemness, tumor mutation burden (TMB), microsatellite instability (MSI), and immune cells infiltration were analyzed using TCGA data. The differential analysis showed that SRI was upregulated in 25 tumor types compared with the normal tissues. Aberrant expression of SRI was able to predict survival in different cancers. Further, the most frequent alteration of SRI genomic was amplification. Moreover, the aberrant SRI expression was related to stemness score, epithelial-mesenchymal-transition (EMT)-related genes, MSI, TMB, and tumor immune microenvironment in various types of cancer. TIMER database mining further found that the SRI expression was significantly correlated with the infiltration levels of various immune cells in certain types of cancer. Intriguingly, the SRI expression was negatively correlated with drug sensitivity of fluorouracil, paclitaxel, docetaxel, and isotretinoin. Our findings highlight the predictive value of SRI in cancer and provide insights for illustrating the role of SRI in tumorigenesis and drug resistance.

## Introduction

Recent statistics showed that cancer has become a worldwide public health issue with an estimated 1,898,160 new cancer cases and 608,570 cancer deaths in 2021 (1). In recent decades, great advances have been achieved in the diagnostics and treatment of cancer, in particular checkpoint blockade-based immunotherapy (2). Currently, reliable predictive biomarkers and new immunotherapy targets have attracted considerable attention among scientists. The SRI gene, which encodes the soluble resistance-related calcium-binding protein (sorcin), is located at chromosome 7q21.12 spanning about 21.9 kb of the human genome (3). Sorcin serves as a calcium-binding protein that is a member of the penta-EF hand (PEF) family (4). Sorcin exists in a soluble form in a low cytoplasmic calcium state but translocates to the membrane to exercise its function in a high cytoplasmic calcium state (5). Classically, sorcin holds a crucial role in the regulation of calcium homeostasis through diverse mechanisms. Sorcin not only directly binds to calcium but also interacts with an L-type calcium channel and cardiac ryanodine receptor-2 to modulate calcium balance (6, 7). The aberrant expression of SRI is reported to be associated with the neurodegenerative diseases, hereditary spherocytosis cells, and women with unexplained infertility (8–10). However, the role of SRI in cancer is receiving gradually more attention.

The previous studies proved that SRI acted as a pro-oncogenic and multidrug resistance gene in certain types of cancers (11–15). The resistance to chemotherapeutic agents is recognized as a major hurdle in cancer therapy. In the past years, MDR1 (ABCB1, P-glycoprotein, and P-gp) and MDR-associated protein 1 (MRP1) were the most extensively investigated resistance proteins in cancer (16, 17). However, SRI as a novel resistance gene has begun to attract substantial attention from scientists (18). Of note, SRI and MDR1 co-localize on the same amplicon and often co-amplify in multidrug-resistant tumor cells (19). The overexpression of sorcin contributed to resistance to many chemotherapy agents, and sorcin knockdown has been found to reverse the multidrug resistance in certain types of cancer (20–22). To date, most of the studies on SRI in tumors are limited by a specific cancer type and many studies have focused on *in vitro* cellular level. Therefore, dissecting the role of SRI in pan-cancer is required.

Our previous findings revealed that SRI promoted the paclitaxel resistance and malignant progression in ovarian cancer (23). Thus, here we postulated that SRI might function as a critical oncogenic, resistant effector in pan-cancer, and played crucial roles in cancer immunity. To uncover the role of SRI in pan-cancer, we systematically integrated multiple databases from bioinformatics point of view. In this study, the expression of SRI was comprehensively investigated in normal tissues and their cancer counterparts using Genotype-Tissue Expression (GTEX), The Cancer Genome Atlas (TCGA), and Oncomine database. Meanwhile, the prognostic value of SRI to predict the survival outcomes was also evaluated. Then, the potential relationships among the SRI expression and clinical features, cancer stemness score, tumor mutation burden (TMB), microsatellite instability (MSI), and infiltrating immune cells were explored in pan-cancer. In addition, the SRI genomic alternations and effect of SRI on the drug sensitivity were determined using the cBioPortal and the CellMiner database. Further, the gene set enrichment analysis (GSEA) was applied to elucidate the biological function of SRI in cancer. Overall, this study highlights the multifaceted role of SRI in pan-cancer, which provides a rationale for targeting SRI as a novel therapeutic strategy.

## Methods

### Data Processing and the SRI Expression Analysis

**Figure 1:**
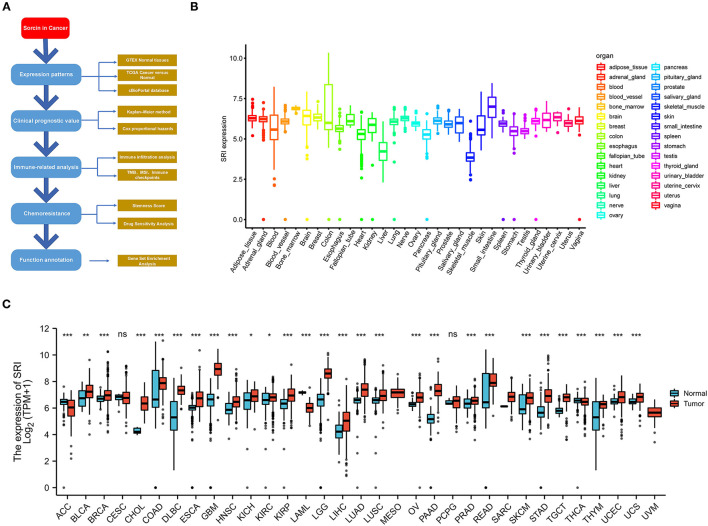
Differential expression of SRI. **(A)** Outline of the workflow for this research. **(B)** The SRI expression in normal tissues from Genotype Tissue-Expression (GTEX) data. **(C)** Differential SRI mRNA expression between 33 The Cancer Genome Atlas (TCGA) cancers and GTEX normal tissues. The red color column represents the cancer samples and the blue color column represents the normal samples. ns, *P* ≥ 0.05; *, *P* < 0.05; **, *P* < 0.01; and ***, *P* < 0.001.

Publicly available transcriptome data of TCGA pan-cancer and the related clinical features were obtained from the UCSC XENA (https://xena.ucsc.edu/). The expression matrices of 31 human normal tissues were downloaded from GTEX web portal (https://www.gtexportal.org/). The strawberry Perl script was developed (version 5.30.0.1, http://strawberryperl.com/) to extract the SRI expression data in 33 TCGA tumor types and GTEX normal tissues. The mRNA level of SRI in the healthy men and women tissues was visualized with “gganatogram” R package. The expression data were log2(TPM) transformed excluding missing data and duplicated values. The differences in the SRI expression between the tumor and normal tissues were examined by the Wilcoxon rank-sum test. The differential expression of SRI mRNA was evaluated in various tumor types using the Oncomine database (www.oncomine.org) (24). All the analyses were conducted using the R version 4.0.2 software (https://www.Rproject.org/). The overall workflow of our study is presented in Figure 1A.

### Correlation of SRI Expression With Survival Prognosis and Clinical Features

The survival and clinical characteristics data were obtained from the UCSC XENA repository. The association between the SRI expression level and survival outcomes, such as overall survival (OS), progression-free interval (PFS), disease-specific survival (DSS), and disease-free survival (DFS), was evaluated using Kaplan–Meier method and Cox proportional hazards model. The “survival,” “survminer,” and “forestplot” packages were employed to draw the Kaplan–Meier and forest plots. The clinical records, such as patents age, tumor stage, and tumor status, were applied to investigate their relationship with the SRI expression. The data representations were performed using “limma” and “ggpubr” R-packages.

### Genomic Alterations SRI in Cancers and the Co-expression Gene Analysis

The SRI gene alternations in TCGA pan-cancer datasets across 10,953 patients were analyzed using cBioPortal database (http://www.cbioportal.org/) (25). The “Oncoprint” and “Cancer Type Summary” modules were used to investigate the genetic alterations of SRI. The “Mutations” module was applied to obtain the mutated site information of SRI. The effect of SRI alternations on the OS and gene mutation co-occurrence analysis were obtained from the “Comparison/Suvival” module. Furthermore, the copy number alterations data, mRNA Expression *Z*-scores data, and protein level *Z*-scores data of SRI in ovarian cancer were downloaded from the TCGA PanCancer Atlas dataset. To investigate the co-expression genes of SRI in ovarian cancer, the TCGA-OV RNA-seq data were accessed using the Linkedomics online tool (http://www.linkedomics.org/login.php/). The top 50 positive and negative correlated genes were selected to construct a co-expression network. The network was visualized in the STRING database (https://string-db.org/).

### Correlation Analysis of SRI Expression With Stemness Score and EMT-Related Genes in Cancers

Cancer stemness was reported to be capable of evaluating by RNA stemness score (RNAss) based on mRNA expression (26). Correlation analysis between SRI expression and RNAss was examined using Spearman rank-based testing. Data were visualized with the R-package “corrplot”. We further determined the correlation between the level of SRI and cancer stem cell marker genes, epithelial-mesenchymal-transition (EMT)-related genes. The heatmaps were generated with the “reshape2” and “RColorBrewer” R-packages.

### Relationship Between SRI Expression and TMB, MSI in Pan-Cancer

Recent studies have revealed that TMB and MSI could become independent biomarkers for immune checkpoint inhibitors response (27, 28). The TCGA pan-cancer mutation data were applied to calculate the TMB scores of each sample. The MSI scores of the TCGA pan-cancer samples were obtained according to a previous study (29). The analyses regarding the correlations between the SRI expression and TMB, MSI were calculated using Spearman's coefficient. The results were displayed as radar plots using the R-package “fmsb.” The tumors with high microsatellite instability are often characterized by a defective DNA mismatch repair system (MMR) system (30). The association between the SRI expression and MMR-related genes was further explored in cancers. The results were visualized as the heatmaps using the “reshape2” and “RColorBrewer” R-packages.

### Association Analysis of the SRI Expression With Tumor Immune Microenvironment in Cancers

The Estimation of Stromal and Immune cells in Malignant Tumors using Expression data (ESTIMATE) algorithm was performed to calculate the ImmuneScore, StromalScore, and ESTIMATEScore using the R package “ESTIMATE” (31). The results were displayed using the R-package “corrplot” using Spearman's rank-based testing. Moreover, we analyzed the correlation of SRI expression with the abundance of various immune cell infiltrates in pan-cancer using the TIMER database (32) (https://cistrome.shinyapps.io/timer/). In addition, a correlation analysis of SRI and immune checkpoint genes was performed. The results were processed using the “reshape2” and “RColorBrewer” R-packages.

### Drug Sensitivity of SRI and GSEA

The NCI-60 compound activity data and the RNA-seq expression profiles were obtained from CellMiner (https://discover.nci.nih.gov/cellminer/home.do) (33). The drugs that were considered FDA approved or in the clinical trials were selected for further analysis. Then, the effect of SRI on the drug sensitivity was analyzed using the “impute,” “limma,” “ggplot2,” and “ggpubr” R package. The GSEA analysis was performed to explore the biological functions of SRI in cancer. The gene sets of Kyoto Encyclopedia of Genes and Genomes (KEGG) and Gene Ontology (GO) signature were obtained from GSEA online (https://www.gsea-msigdb.org/gsea/downloads.jsp). The GSEA analysis was processed with the R-packages “enrichplot,” “org.Hs.eg.db,” “clusterProfiler,” “DOSE,” and “limma”.

### Sorcin Expression in the Ovarian Cancer Sphere Cells

The cell culture and sphere formation assays were performed according to the previously described method (34). The sorcin expression in the ovarian cancer sphere cells was evaluated using a western blot. Anti-SRI antibody (ab71983) was purchased from Abcam (Cambridge, UK).

### Statistical Analysis

A log-rank test was applied in the Kaplan–Meier survival curves. Hazard ratio (HR) was determined by a Cox proportional hazard regression model. The correlation analysis was executed by Spearman's rank test. The statistical analyses were performed using the R version 4.0.2 software (R Foundation for Statistical Computing, Vienna, Austria) or GraphPad Prism 8 (San Diego, CA, USA). Results with *P* < 0.05 were considered statistically significant (^*^P < 0.05, ^**^P < 0.01, ^***^P < 0.001, and ^****^P < 0.0001).

## Results

### Expression Levels of SRI in Normal Tissues and Pan-Cancer

To gain insights into the expression pattern of SRI in the human normal tissues, the SRI expression in tissue physiological state was investigated according to GTEX dataset. SRI was highly expressed in the small intestine, bone marrow, brain, and breast tissues, while the skeletal muscle and liver tissues expressed low levels of SRI (Figure 1B). The SRI expression abundances of various tissues in men and women are displayed in Supplementary Figures 1A,B. Overall, no gender difference was observed in the mRNA expression levels of SRI (Supplementary Figure 1C).

To further explore the SRI expression in human cancers, the SRI expression in various types of cancers was analyzed using the RNA-seq data of TCGA database (Figure 1C). The aberrant expression of SRI was detected in 28 types of cancer except for those cancers where no normal tissue data were available. The SRI expression was significantly higher in bladder urothelial carcinoma (BLCA), breast invasive carcinoma (BRCA), cholangiocarcinoma (CHOL), colon adenocarcinoma (COAD), lymphoid neoplasm diffuse large B-cell lymphoma (DLBC), esophageal carcinoma (ESCA), glioblastoma multiforme (GBM), head and neck squamous cell carcinoma (HNSC), kidney chromophobe (KICH), kidney renal clear cell carcinoma (KIRC), kidney renal papillary cell carcinoma (KIRP), brain lower grade glioma (LGG), liver hepatocellular carcinoma (LIHC), lung adenocarcinoma (LUAD), lung squamous cell carcinoma (LUSC), ovarian serous cystadenocarcinoma (OV), pancreatic adenocarcinoma (PAAD), prostate adenocarcinoma (PRAD), rectum adenocarcinoma (READ), skin cutaneous melanoma (SKCM), stomach adenocarcinoma (STAD), testicular germ cell tumors (TGCT), thymoma (THYM), uterine corpus endometrial carcinoma (UCEC), and uterine carcinosarcoma (UCS). In contrast, the SRI levels were significantly downregulated in adrenocortical carcinoma (ACC), acute myeloid leukemia (LAML), and thyroid carcinoma (THCA). Aside from this, the higher level of SRI was reconfirmed in the brain, bladder, esophageal, gastric, head and neck, kidney, liver, melanoma, myeloma, pancreatic, and prostate cancer compared with the normal tissues using ONCOMINE database (Supplementary Figure 1D).

### Prognostic Value of SRI in Pan-Cancer

**Figure 2:**
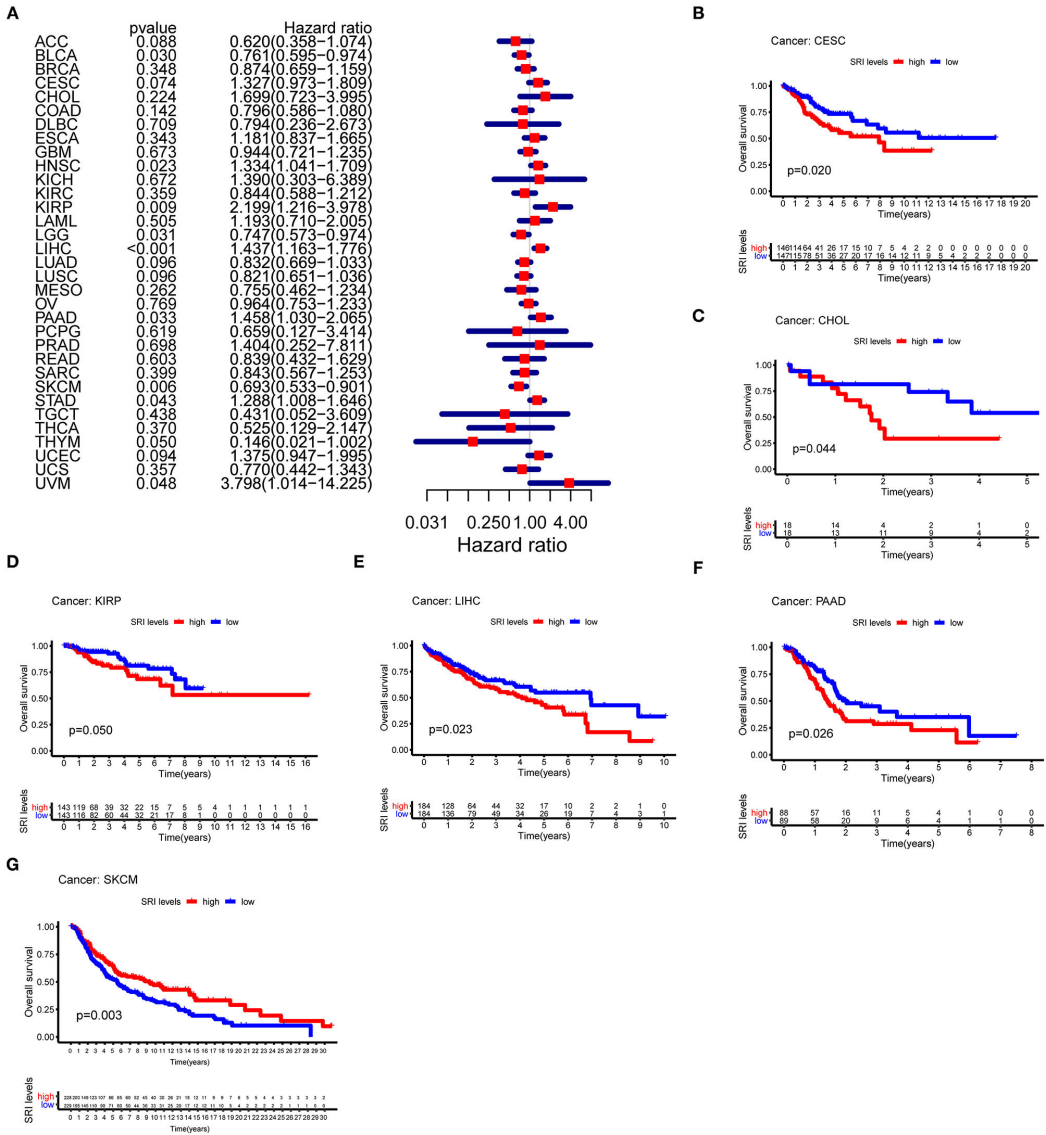
The Cox proportional hazards model and Kaplan–Meier analysis of overall survival (OS) time by the SRI expression. **(A)** A forest plot of the associations of SRI expression and OS in 33 types of tumor. **(B–G)** Kaplan–Meier analysis of the correlations between the SRI expression and OS. A red line represents high SRI expression, and the blue lines represent low SRI expression.

**Figure 3:**
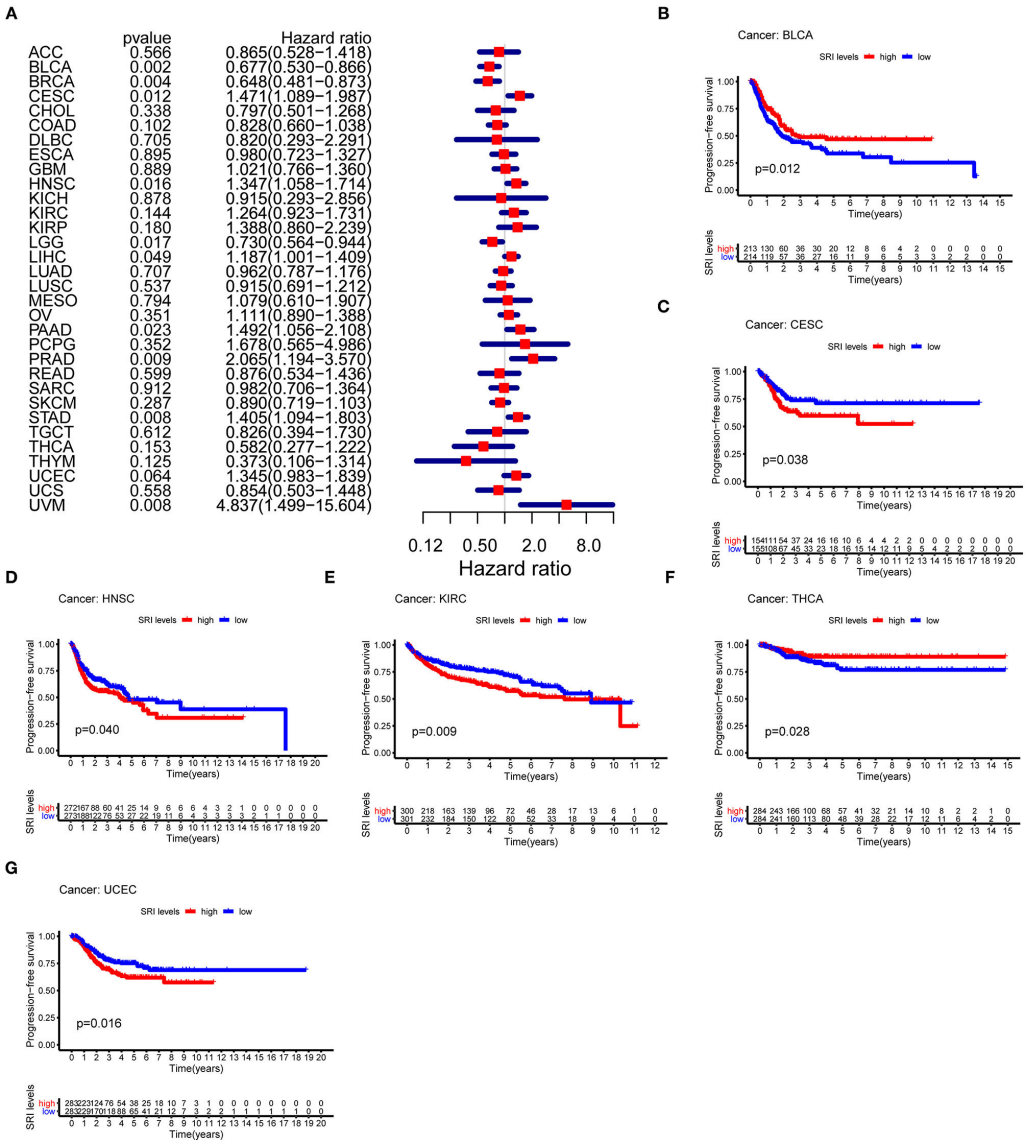
The Cox proportional hazards model and Kaplan–Meier analysis of progression-free survival (PFS) time by the SRI expression. **(A)** A forest plot of the associations of SRI expression and PFS in 33 types of tumor **(B–G)** Kaplan–Meier analysis of the correlations between the SRI expression and PFS. A red line represents the high SRI expression, and the blue lines represent the low SRI expression.

**Figure 4:**
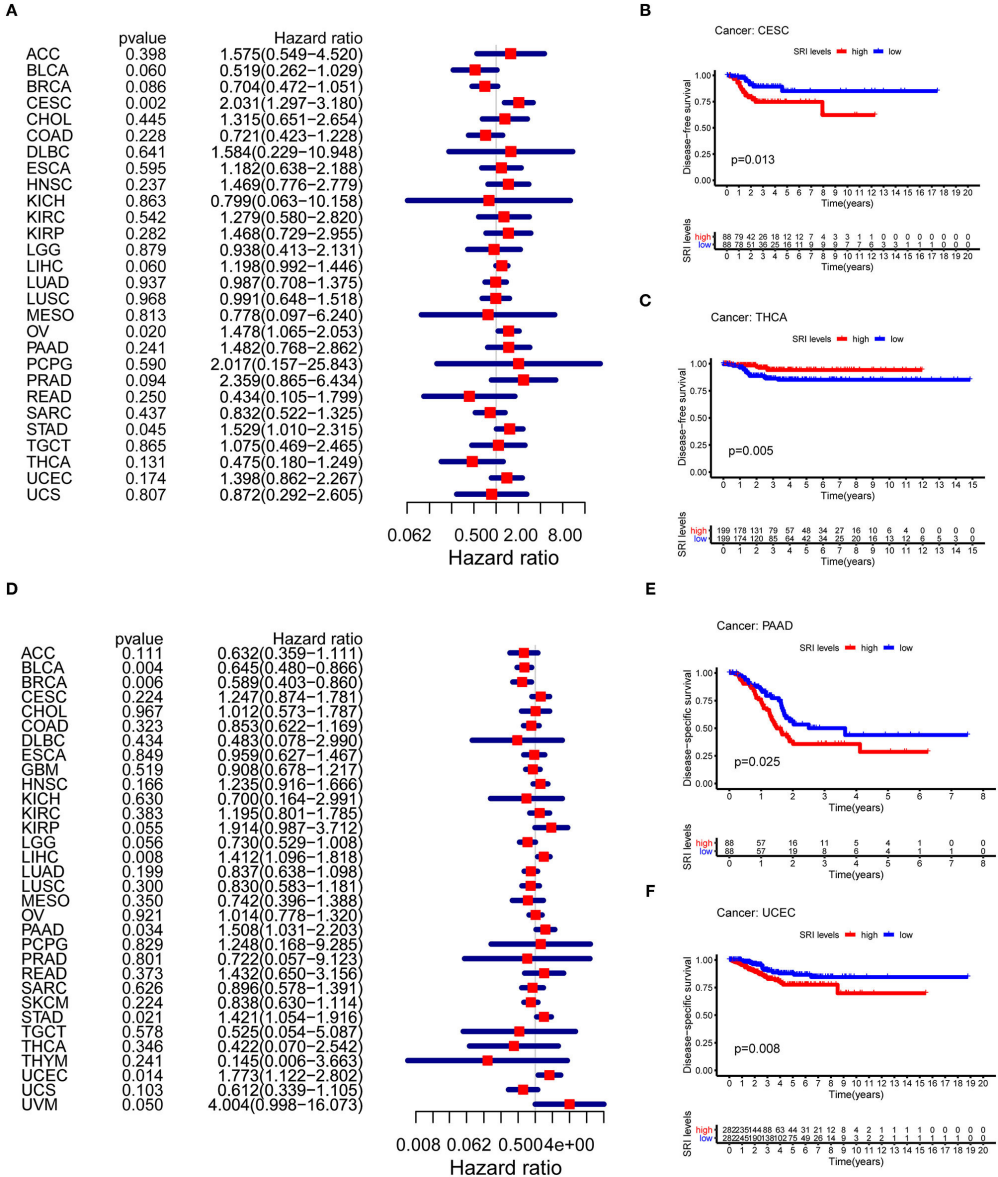
The Cox proportional hazards model and Kaplan–Meier analysis of disease-free survival (DFS) time and disease-specific survival (DSS) by the SRI expression. **(A)** A forest plot of the associations of SRI expression and DFS in 33 types of tumor **(B,C)** Kaplan–Meier analysis of the correlations between the SRI expression and DFS. A red line represents the high SRI expression, and the blue lines represent the low SRI expression. **(D)** A forest plot of the associations of SRI expression and DSS in 33 types of tumor **(E,F)** Kaplan–Meier analysis of the correlations between the SRI expression and DSS. A red line represents high the SRI expression, and the blue lines represent the low SRI expression.

To further explore the prognostic value of SRI in pan-cancer, the association between the SRI expression level and survival of patients was evaluated using the Cox proportional hazards model and the Kaplan-Meier analysis. The results from a Cox proportional hazards regression model revealed that the SRI expression levels were correlated with OS in BLCA (*P* = 0.030), HNSC (*P* = 0.023), KIRP (*P* = 0.009), LIHC (*P* < 0.001), LGG (*P* = 0.001), PAAD (*P* = 0.033), SKCM (*P* = 0.006), STAD (*P* = 0.043), THYM (*P* = 0.05), and uveal melanoma (UVM) (*P* = 0.048) (Figure 2A). SRI served as a high-risk factor for HNSC, KIRP, LIHC, PAAD, STAD, and UVM, while it acted as a low-risk gene for BLCA, LGG, SKCM, and THYM. Furthermore, the Kaplan–Meier survival analysis demonstrated that the high level of SRI predicted poor OS in cervical squamous cell carcinoma and endocervical adenocarcinoma (CESC) (Figure 2B, *P* = 0.020), CHOL (Figure 2C, *P* = 0.044), KIRP (Figure 2D, *P* = 0.050), LIHC (Figure 2E, *P* = 0.023), and PAAD (Figure 2F, *P* = 0.026). While low expression of SRI was correlated with the shortened OS in SKCM (Figure 2G, *P* = 0.003). Regarding the PFS, the high SRI level represented an adverse factor in CESC (*P* = 0.012), HNSC (*P* = 0.016), LIHC (*P* = 0.049), PAAD (*P* = 0.023), PRAD (*P* = 0.009), STAD (*P* = 0.008), and UVM (*P* = 0.008), while the high SRI level was considered as a favorable factor in BLCA (*P* = 0.002), BRCA (*P* = 0.004), and LGG (*P* = 0.017) (Figure 3A). The Kaplan–Meier curves for PFS revealed a correlation between the high SRI expression level and poor survival time in the patients with CESC (Figure 3C, *P* = 0.038), HNSC (Figure 3D, *P* = 0.040), KIRC (Figure 3E, *P* = 0.009), and UCEC (Figure 3G, *P* = 0.016). The patients with high SRI expression had significantly longer PFS than the patients with low expression in BLCA (Figure 3B, *P* = 0.012) and THCA (Figure 3F, *P* = 0.028). Regarding the associations between the SRI expression and DFS, the forest plots showed high SRI expression predicted poor DFS in CESC (*P* = 0.002), OV (*P* = 0.020), and STAD (*P* = 0.040) (Figure 4A). Significant relationships between the SRI expression and DFS were observed in CESC (Figure 4B, *P* = 0.013) and THCA (Figure 4C, *P* = 0.005) by the Kaplan–Meier survival analysis. Furthermore, SRI exhibited a significant prognostic value in BLCA (*P* = 0.004), BRCA (*P* = 0.006), LIHC (*P* = 0.008), PAAD (*P* = 0.034), STAD (*P* = 0.021), UCEC (*P* = 0.014), and UVM (*P* = 0.05) in a Cox proportional hazards regression model for DSS (Figure 4D). The Kaplan–Meier survival analysis found that the PAAD (Figure 4E, *P* = 0.025) and UCEC (Figure 4F, *P* = 0.008) patients with high expression of SRI had shortened DSS. Additionally, the survival analysis of SRI in some types of cancer was validated in the GEO database (Supplementary Figure 2).

### Correlation Between the SRI Expression and Clinicopathological Phenotypes in Cancers

**Figure 5:**
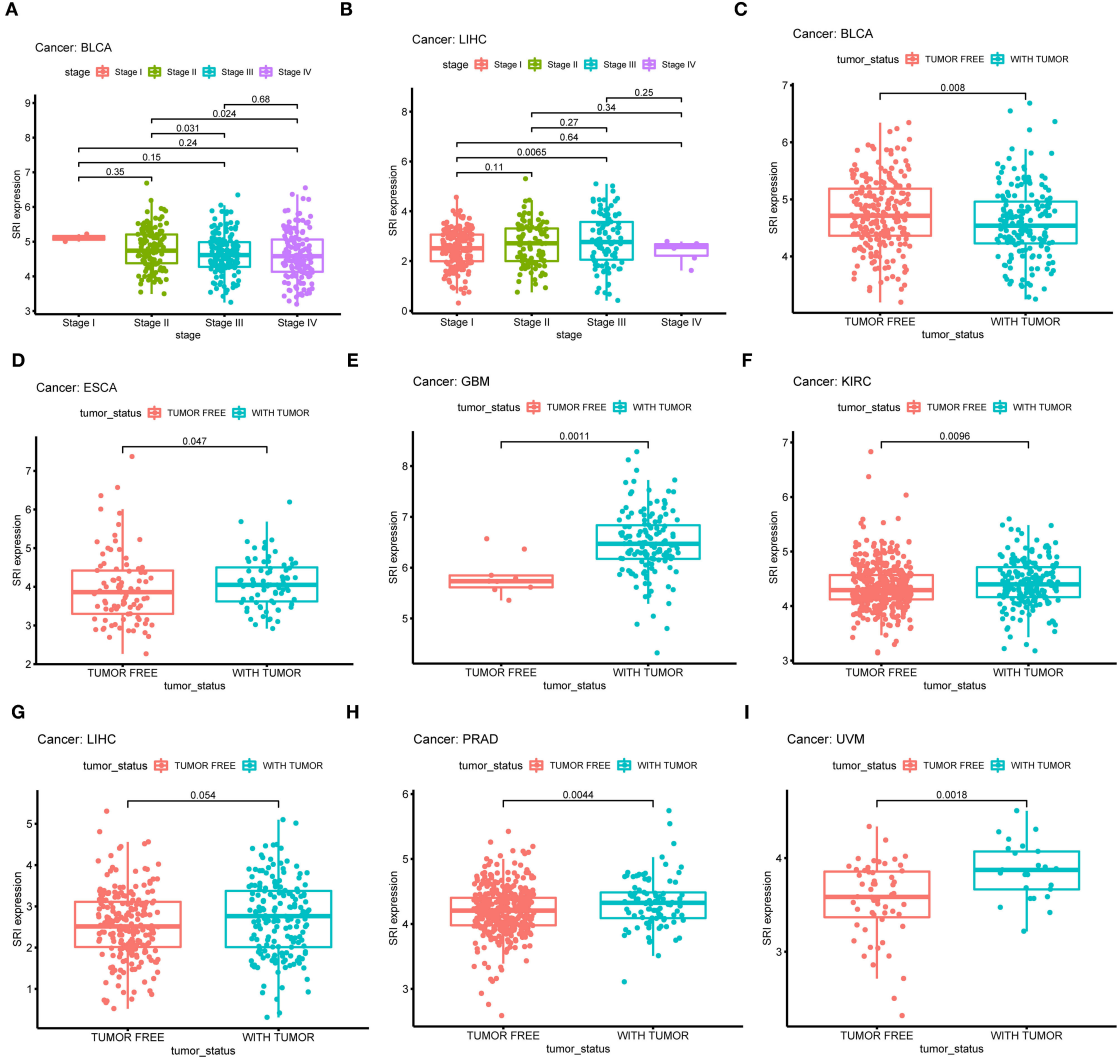
Correlation between SRI expression and stage in **(A)** bladder urothelial carcinoma (BLCA), **(B)** liver hepatocellular carcinoma (LIHC). Correlation between SRI expression and tumor status in **(C)** bladder urothelial carcinoma (BLCA), **(D)** esophageal carcinoma (ESCA), **(E)** glioblastoma multiforme (GBM), **(F)** kidney renal clear cell carcinoma (KIRC), **(G)** liver hepatocellular carcinoma (LIHC), **(H)** prostate adenocarcinoma (PRAD), and **(I)** uveal melanoma (UVM).

Next, the correlations between the SRI expression and the clinicopathological features of patients were investigated in pan-cancer. Patients with age ≥65 years had higher expression of SRI in KIRC (Supplementary Figure 3A; *P* = 0.011) and PRAD (Supplementary Figure 3B; *P* = 0.012). No significant difference was observed between the SRI expression and age in other cancers. We further compared the differential mRNA level of SRI in different tumor stages. SRI expression tended to decrease from stage I to stage IV in BLCA (Figure 5A). In contrast, the high SRI expression tended to associate with the advanced tumor stages in LIHC (Figure 5B). The difference between stage I and IV tumors was not statistically significant in LIHC, one reason that might be responsible is the small patient numbers in the advanced stage. Tumor status after treatment was closely associated with disease recurrence. We found that the high level of SRI was significantly correlated with-tumor status in ESCA (Figure 5D), GBM (Figure 5E), KIRC (Figure 5F), LIHC (Figure 5G), PRAD (Figure 5H), and UVM (Figure 5I). While a high expression of SRI was significantly related to the tumor-free status in BLCA (Figure 5C).

### Genetic Alteration Analysis of SRI in Pan-Cancer

**Figure 6:**
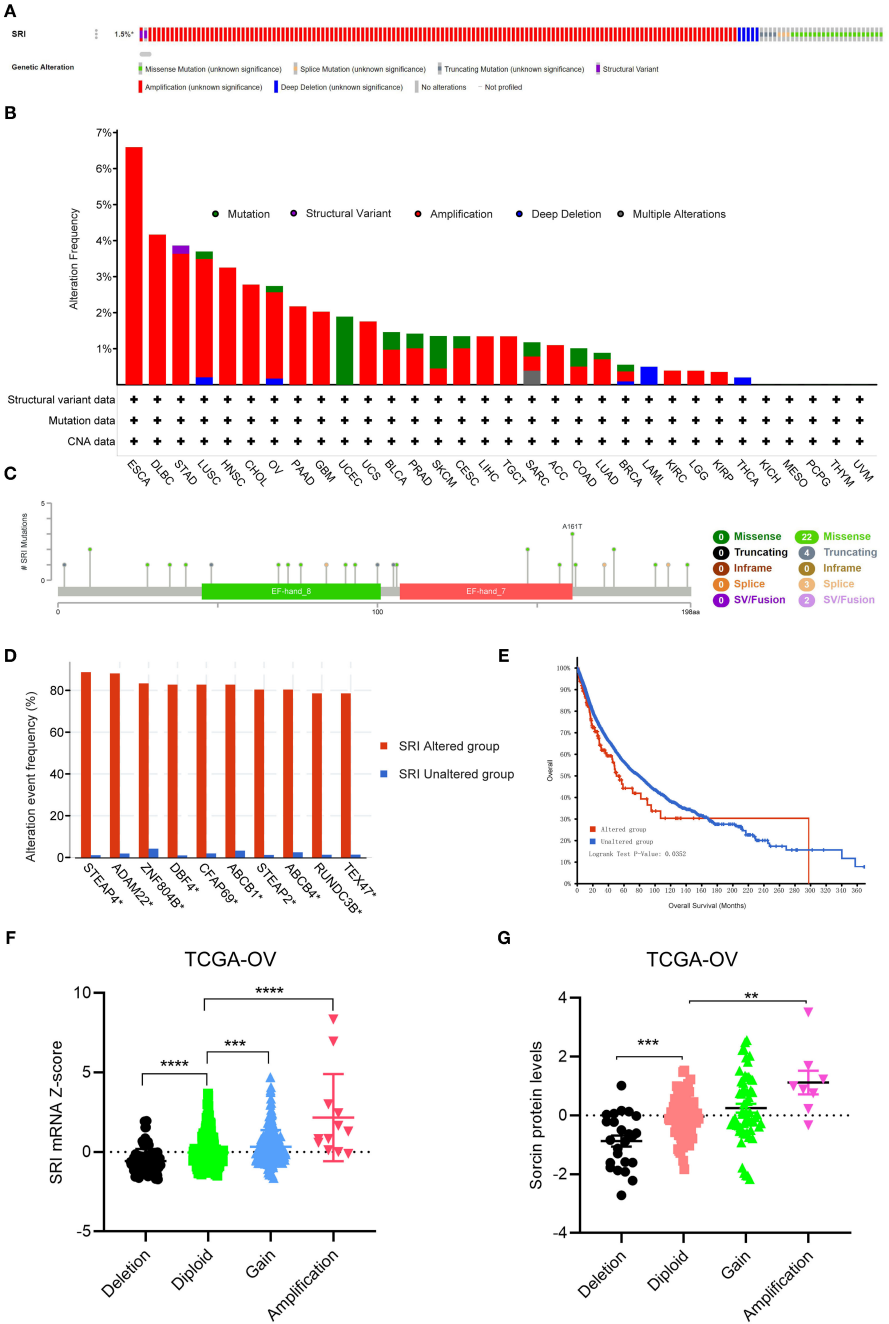
The genetic alterations of SRI in TCGA pan-cancer. **(A)** OncoPrint summary of the alterations on SRI in TCGA pan-cancer datasets. **(B)** Summary of the alteration frequency of SRI derived from structural variant, mutations, and copy-number alterations data in TCGA pan-cancer datasets. **(C)** The mutation types, number, and sites of the SRI genetic alterations. **(D)** The analysis of gene mutation co-occurrence comparing the altered group and unaltered group of SRI. **(E)** Kaplan–Meier overall survival of TCGA pan-cancer cohort with altered or unaltered SRI. **(F)** Association of the SRI copy number alterations with its mRNA expression in the TCGA ovarian cancer cohort. ***P < 0.001, ****P < 0.0001, and by one-way ANOVA followed by Tukey's test. **(G)** Association of SRI copy number alterations with its protein expression in the TCGA ovarian cancer cohort. **P < 0.01, ***P < 0.001, and by one-way ANOVA followed by Tukey's test.

Next, the cBioPortal database was applied to investigate the genetic alterations of SRI in the TCGA pan-cancer datasets. As shown in Figure 6A, the SRI gene was altered in 168 patients with cancer which accounted for only 1.5% across 10,953 samples. Regarding the SRI alterations in different cancer types (Figure 6B), the most frequent alterations of SRI gene were amplification in ESCA, DLBC, STAD, LUSC, HNSC, CHOL, OV, PAAD, GBM, UCS, BLCA, PRAD, CESC, LIHC, TGCT, ACC, LUAD, BRCA, KIRC, LGG, and KIRP. Patients with UCEC, SKCM harbored High frequency of SRI mutations was observed in UCEC, SKCM, while patients with LAML and THCA harbored high frequency of SRI deep deletion of SRI gene was the most frequent mutation type in LAML and THCA. The mutation types, number, and sites of the SRI genetic alterations are displayed in Figure 6C. The results showed that the missense mutations were the major mutation type of SRI. Intriguingly, co-occurrence of STEAP4, ADAM22, ZNF804B, DBF4, CFAP69, ABCB1, STEAP2, ABCB4, RUNDC3B, and TEX47 alterations was observed with the SRI alterations (Figure 6D). While the patients with SRI alterations represented only a small part, the patients in the SRI genetic altered group had poorer OS than those in the SRI unaltered group (Figure 6E). To explore whether the SRI amplification had an influence on its mRNA and protein level, the copy number alterations data and expression data of SRI were acquired in TCGA ovarian cancer. The results indicated that SRI was amplified along with the significantly high mRNA and protein level in TCGA-OV cohort (Figures 6F,G). Regarding the co-expression genes of SRI, the co-expression analysis was conducted using the Linkedomics database in the TCGA ovarian cancer dataset. The results are presented in Supplementary Table 1. The top 50 positive and negative co-expression genes of SRI were visualized in the STRING database (Supplementary Figure 4).

### Correlation Among the SRI Expression and Stemness Score, EMT-Related Genes, MSI, TMB, and MMR-Related Genes in Pan-Cancer

**Figure 7:**
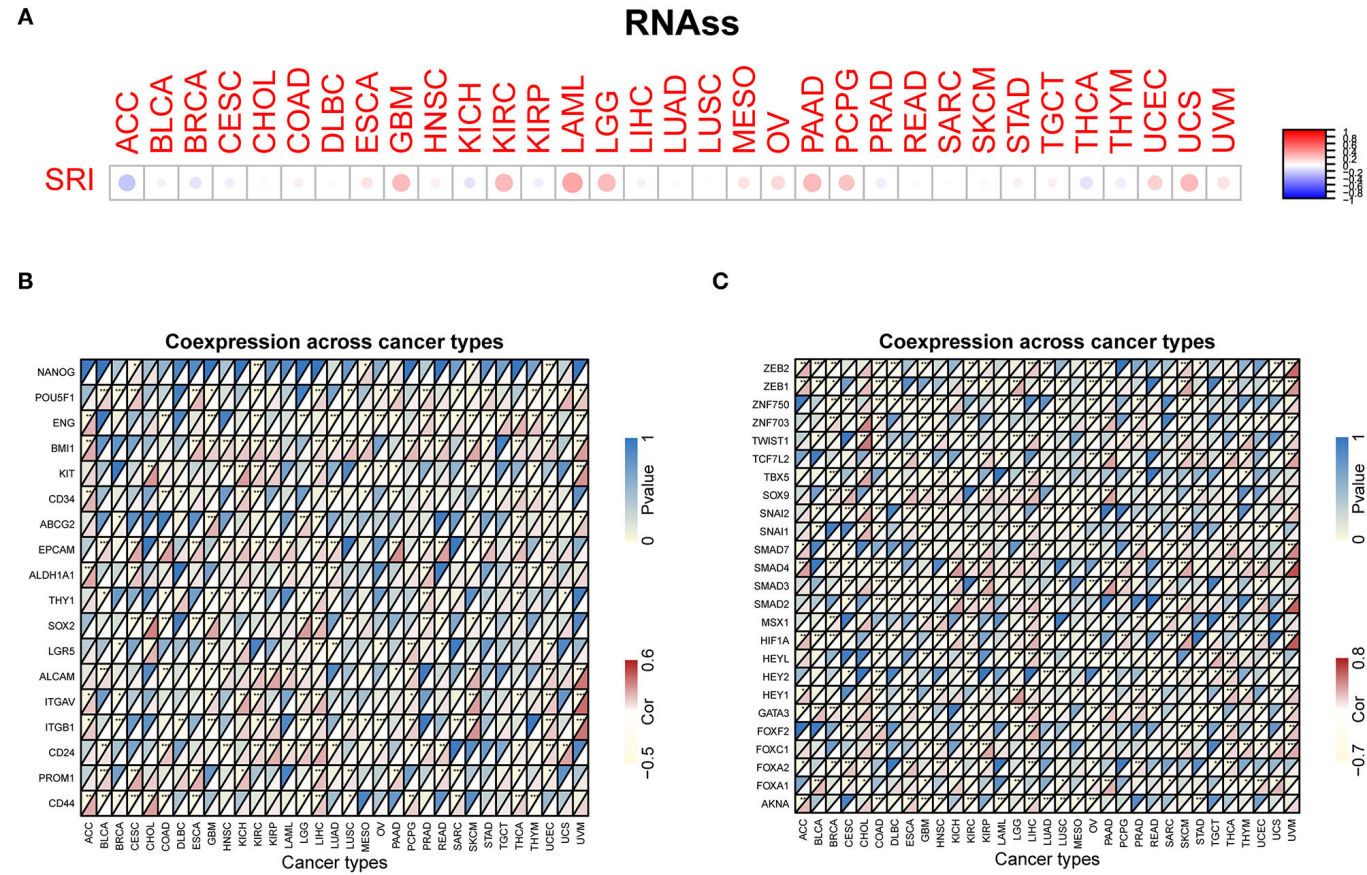
Correlation of the SRI expression with stemness and EMT-related genes in cancers. **(A)** The SRI expression associated with RNA stemness score (RNAss) in different cancers. The red dots indicate a positive correlation and the blue dots indicate a negative correlation. RNAss, RNA stemness score; a heatmap indicating the correlation between the SRI expression and cancer stem cell markers expression **(B)**, EMT-related genes expression **(C)**. For each pair, the top left triangle represents the *P*-value, and the bottom right triangle represents the correlation coefficient *P < 0.05, **P < 0.01, and ***P < 0.001.

**Figure 8:**
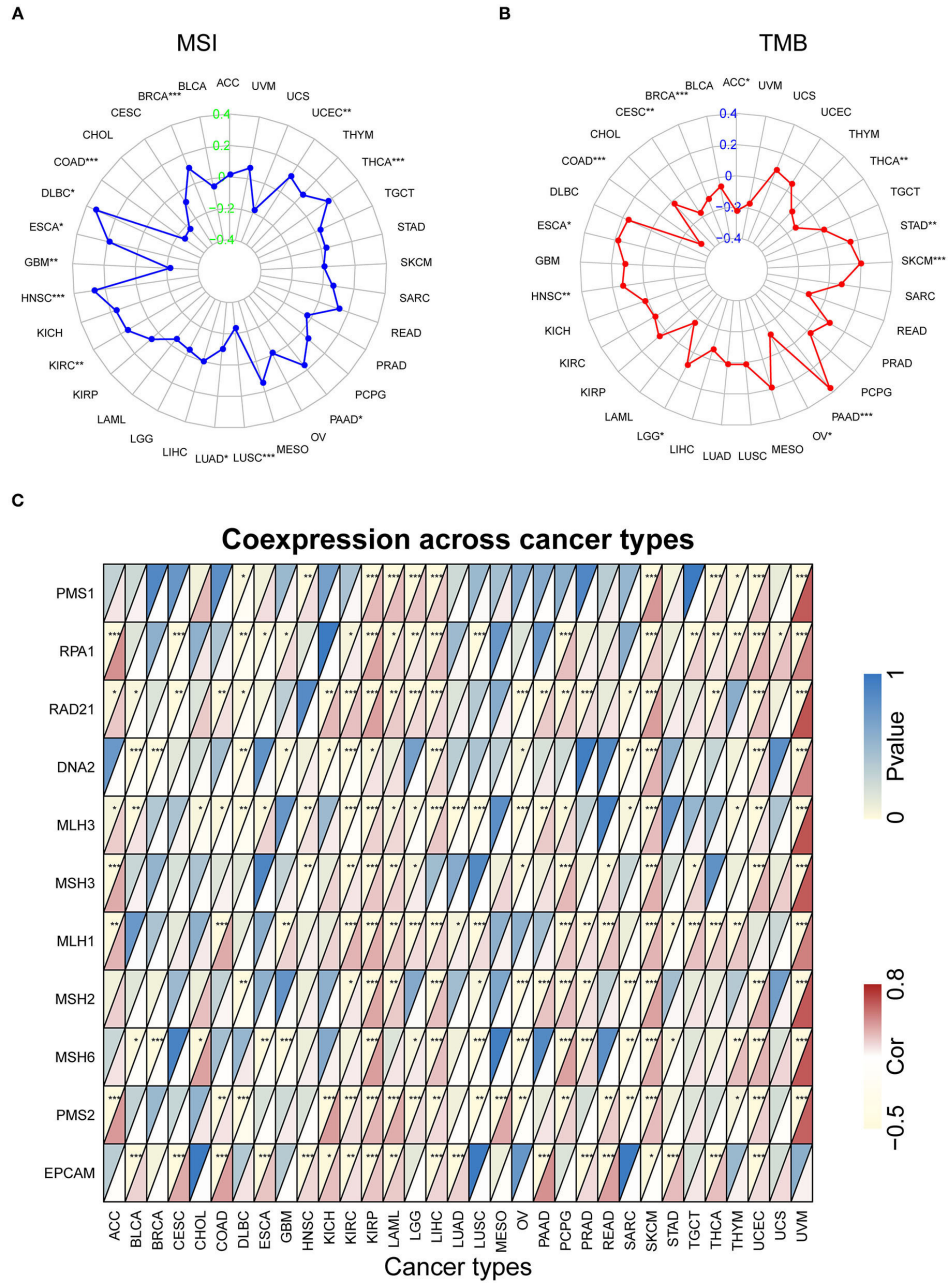
The correlation of SRI expression with microsatellite instability (MSI), tumor mutational burden (TMB), and mismatch repair (MMR) genes. **(A)** Radar map of correlation between the SRI expression and MSI. **(B)** Radar map of correlation between the SRI expression and TMB. **(C)** A heatmap indicating the correlation between the SRI expression and MMR genes. For each pair, the top left triangle indicates the *P*-value, and the bottom right triangle indicates the correlation coefficient *P < 0.05, **P < 0.01, and ***P < 0.001.

Our previous work revealed that SRI promoted the stemness and EMT process in ovarian cancer (35), we, therefore, wanted to investigate the association of SRI expression with stemness score and EMT-related genes in cancers. First, the sorcin expression was upregulated in the OVCAR-3 and SKOV-3 spheres cells compared with the respective adherent cells (Supplementary Figure 5). Correlation analysis indicated that the SRI expression was positively associated with the RNAss in GBM, KIRC, LAML, LGG, OV, PADD, pheochromocytoma and paraganglioma (PCPG), UCEC, and UCS. While a negative relationship between the SRI level and RNAss was observed in ACC (Figure 7A). Figure 7B depicts the correlations between the SRI expression and 18 tumor stem cell markers. More than 10 cancer stem cell markers expression were significantly positively correlated with the SRI levels in GBM, KIRC, LGG, LIHC, SKCM, THCA, and UCEC. Furthermore, a heatmap showed a positive correlation between the SRI expression and 25 EMT-related genes (Figure 7C). The expression of over 15 EMT-related genes was positively associated with the SRI expression in COAD, GBM, KIRC, LIHC, and OV. The correlations among the TMB, MSI, and SRI expression were further analyzed in cancers. The results demonstrated that the SRI expression was significantly associated with increased MSI in BRCA, DLBC, ESCA, HNSC, KIRC, PAAD, THCA, and UCEC, while the SRI expression was negatively associated with MSI in COAD, GBM, LUAD, and LUSC (Figure 8A). In addition, SRI was positively correlated with TMB in ESCA, HNSC, LGG, PAAD, SKCM, and STAD, while a negative correlation between the SRI expression and TMB was found in ACC, BRCA, CESC, COAD, OV, and THCA (Figure 8B). As shown in Figure 8C, the SRI expression was significantly positively correlated with the MMR-related genes level in most of the tumors, especially in KIRP, LIHC, SKCM, UCEC, and UVM.

### Correlation of the SRI Expression With Tumor Immune Microenvironment

**Figure 9:**
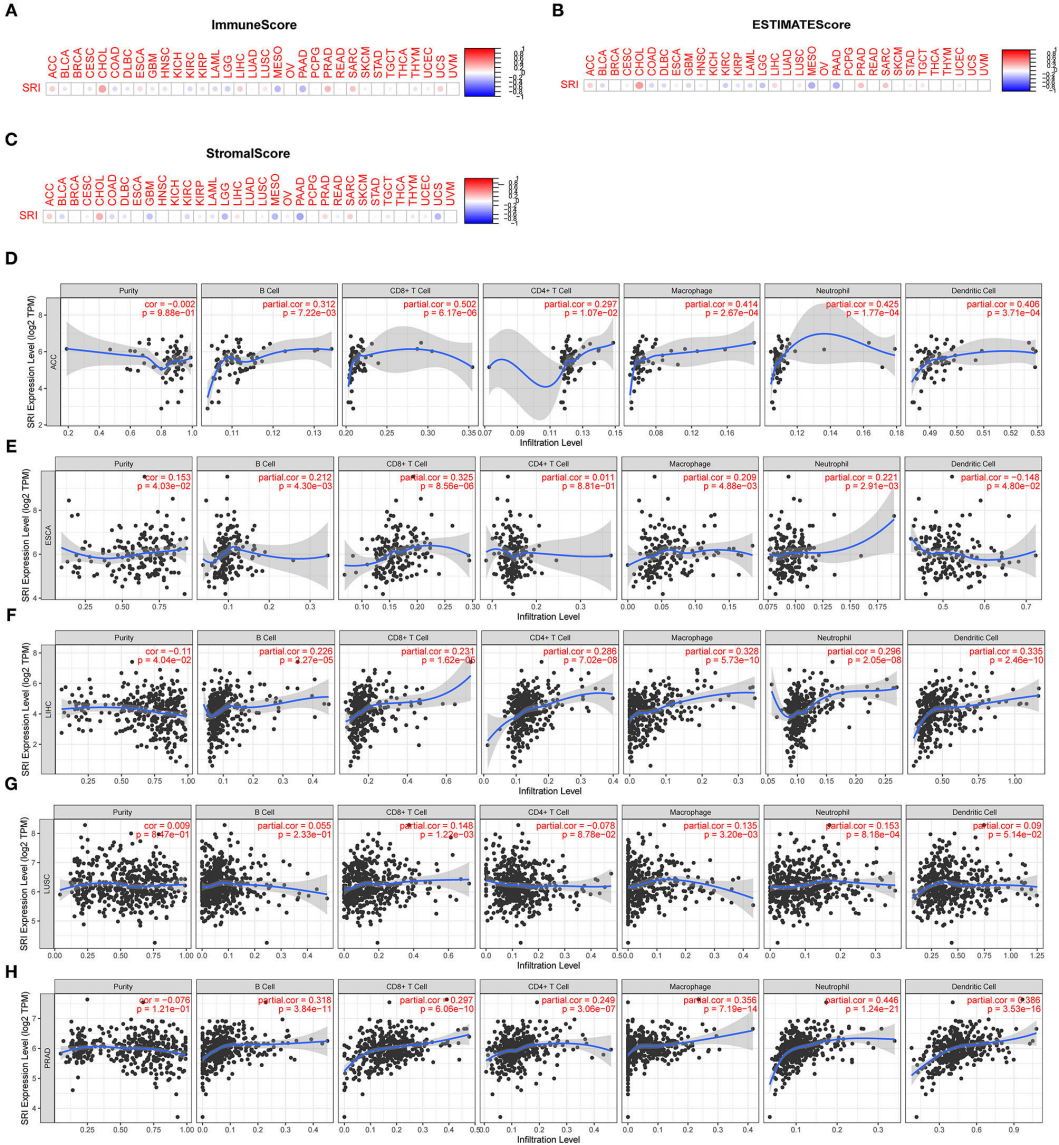
Correlation between the SRI gene expression and tumor immune microenvironment in TCGA. **(A–C)** The SRI expression associated with ImmuneScore, ESTIMATEScore, and StromaScore in different cancers. The red dots indicate a positive correlation and the blue dots indicate a negative correlation. **(D–H)** Correlation of the SRI expression with immune infiltration level in adrenocortical carcinoma (ACC), ESCA, LIHC, lung squamous cell carcinoma (LUSC), and PRAD in TIMER database.

Presently, the predictive role of SRI in tumor immune microenvironment has received little attention. Here, the association of SRI expression with the tumor immune microenvironment was evaluated according to the ESTIMATE algorithm and TIMER database. Our findings showed that the SRI expression had a positive correlation with the immune scores and estimate scores in ACC, ESCA, CHOL, LIHC, PRAD, and SARC (sarcoma). A strong negative correlation between the SRI and immune scores and estimate scores was found in LGG, MESO, and PAAD (Figures 9A,B). In addition, the correlation analyses revealed that the stromal scores were negatively correlated with the SRI expression in GBM, LGG, MESO, PADD, and UCS (Figure 9C). Then, the relationship between the SRI expression and immune cells infiltration was investigated in pan-cancer. The results indicated that the SRI expression was significantly associated with tumor purity in five cancer types. Furthermore, the SRI expression was significantly correlated with the infiltration levels of B cells in 14 cancer types, CD8+T cells in 14 cancer types, CD4+T cells in 13 cancer types, the macrophages in 21 cancer types, the neutrophils in 16 cancer types, and the dendritic cells in 12 cancer types (Figures 9D–H; Supplementary Figures 6, 7). Combined results from the correlation analysis and TIMER database, the SRI expression was strongly correlated with the immune infiltrating level in ACC, ESCA, LIHC, LUSC, and PRAD. The correlation between the immune checkpoint genes expression and SRI expression was explored. The results demonstrated that the most immune checkpoint genes were positively correlated with the SRI expression, especially in LIHC, PRAD, UVM, and ACC (Supplementary Figure 8), which suggested that the high level of SRI might mediate immune escape.

### Drug Sensitivity Analysis of SRI

**Figure 10:**
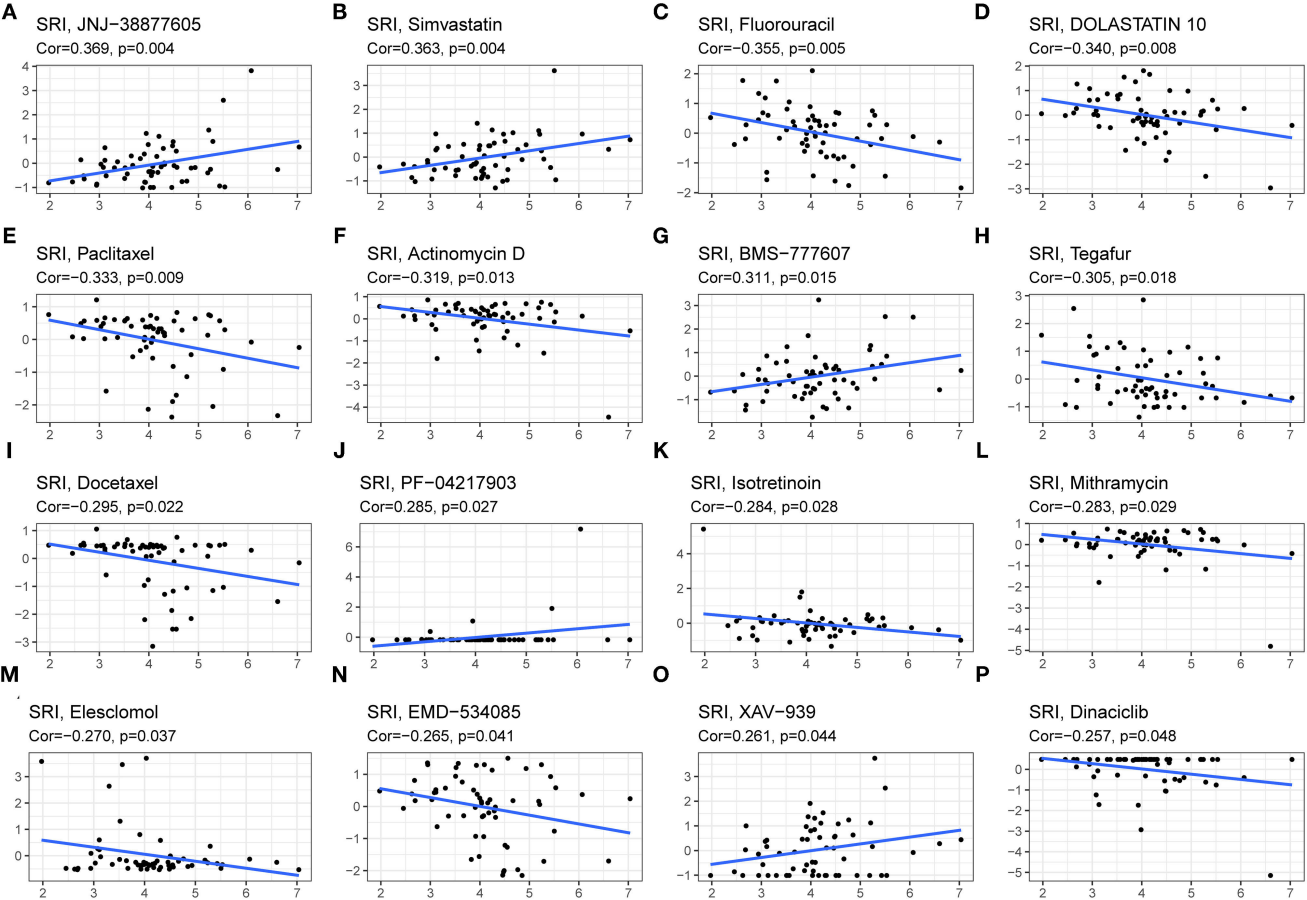
The drug sensitivity analysis of SRI. The SRI expression was positively associated with drug sensitivity of JNJ-38877605 **(A)**, Simvastatin **(B)**, BMS-777607 **(G)**, PF-04217903 **(J)**, and XAV-939 **(O)**. The SRI expression was negatively associated with drug sensitivity of Fluorouracil **(C)**, DOLASTATIN 10 **(D)**, Paclitaxel **(E)**, Actinomycin D **(F)**, Tegafur **(H)**, Docetaxel **(I)**, Isotretinoin **(K)**, Mithramycin **(L)**, Elesclomol **(M)**, EMD-534085 **(N)**, and Dinaciclib **(P)**. *X*-axis represents the SRI expression level and *Y*-axis represents the scores of drug sensitivity.

Since the drug resistance role of SRI in cancer has been gradually valued, we further investigated the potential correlation analysis between the drug sensitivity and SRI expression using the CellMiner™ database. Our results indicated that the SRI expression was positively related to JNJ-38877605, Simvastatin, BMS-777607, PF-04217903, and XAV-939 sensitivity (Figures 10A,B,G,J,O). Notably, the SRI expression was negatively correlated with the drug sensitivity of fluorouracil, dolastatin 10, paclitaxel, actinomycin D, tegafur, docetaxel, isotretinoin, mithramycin, elesclomol, EMD-534085, and dinaciclib (Figures 10C–F,H,I,K–N,P).

### Biological Function of SRI in Cancer

**Figure 11:**
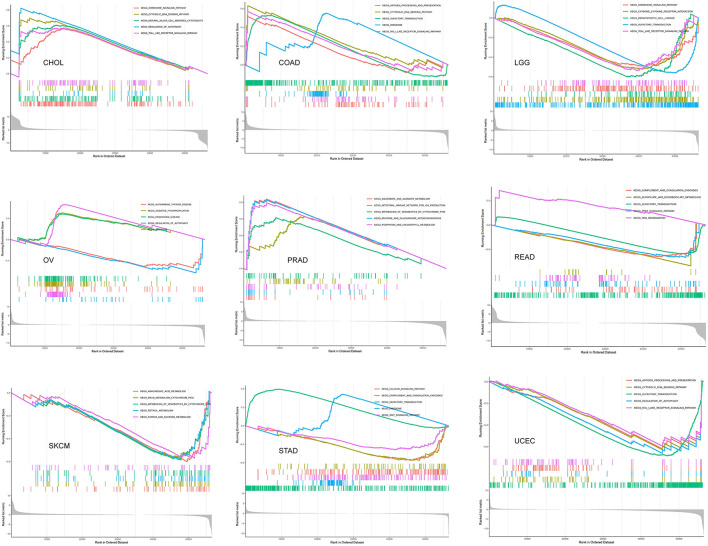
The gene set enrichment analysis (GSEA) analysis in Kyoto Encyclopedia of Genes and Genomes (KEGG) signature of cholangiocarcinoma (CHOL), colon adenocarcinoma (COAD), brain lower-grade glioma (LGG), ovarian serous cystadenocarcinoma (OV), PRAD, rectum adenocarcinoma (READ), skin cutaneous melanoma (SKCM), stomach adenocarcinoma (STAD), and uterine corpus endometrial carcinoma (UCEC). The different colors curves represent the different functions or pathways. The peaks on the upward curve indicate positive regulation by SRI and peaks on the downward curve indicate negative regulation by SRI.

The GSEA was then performed to explore the main biological process affected by SRI in cancer. In the KEGG pathway gene set analysis, our results suggested that SRI positively regulated the immune-related pathways in CHOL, COAD, PRAD, such as natural cell-mediated cytotoxicity, antigen processing, and presentation. SRI was positively enriched in the metabolism-related pathways of OV and PRAD, such as oxidative phosphorylation, ascorbate and aldarate metabolism, and chlorophyll metabolism. In contrast, SRI was negatively enriched in the glyoxylate and dicarboxylate metabolism and drug metabolism cytochrome P450 in READ and SKCM. In addition, SRI was identified as a negative regulator for olfactory transduction, autophagy, WNT signaling pathway in LGG, READ, OV, UCEC, and STAD (Figure 11). The GO results of GSEA analysis of SRI in pan-cancer are displayed in Supplementary Figures 9, 10. In ACC, CESC, HNSC, LIHC, LAML, and SKCM, the SRI expression showed positive enrichment in the gene regulatory mechanisms, such as gene silencing, alternative mRNA splicing, transcription activator activity, and methylation CPG binding. Several immune response pathways, such as immune response regulating cell surface receptor signaling, regulation of immune effector process, and response to interleukin 12 were positively correlated to the SRI expression in STAD, CHOL, and SARC. Furthermore, a negative enrichment among the cell cycle G1/S transition, regulation of epidermal cell growth, and SRI expression was observed in OV, LUSC, PRAD, UCS, PCPG, READ, and ESCA.

## Discussion

In the present study, high expression of SRI in multiple cancers was observed and its dysregulation could predict worse prognosis in the patients with cancer, which indicated that SRI could serve as a robust prognostic factor among a variety of cancers. SRI exerts its function *via* the regulation of stemness, MSI, TMB, tumor immune microenvironment, and drug resistance. Sorcin, one of the most abundant calcium-binding proteins, plays an essential role in excitable cells, such as neurons and cardiomyocytes (36). Regarding the SRI expression in normal tissues, our research revealed that the small intestine, brain tissues, and bone marrow had a high expression abundance of SRI, which was consistent with the reports of PaxDb database (https://pax-db.org/) (18). Cytosolic calcium (Ca^2+^), one of the most fascinating cell signaling, organizes the diverse physiological activities from cell division, cellular motility to cell death (37). As an overexpressed Ca^2+^ binding protein, sorcin exquisitely controls the intracellular calcium content and exchange under normal physiological conditions. Multiple mechanisms are reported in the regulation of calcium balance affected by sorcin, some of which depend on the interaction with other calcium-related proteins (38). For a long time, substantial evidence suggests that Ca^2+^ signaling is implicated in the cancer cells' uncontrolled proliferation, angiogenesis, immune surveillance, and drug resistance (39). The previous studies have reported overexpression of SRI in the cell lines of gastric cancer, colorectal cancer, and breast cancer (13–15). Meanwhile, the high expression level of SRI was observed in the resistance cells of ovarian cancer, myeloma, lung cancer, leukemia, and nasopharyngeal carcinoma (20, 40–44). In this study, the high expression of SRI was verified in 25 tumor types compared with the normal tissues. Until now, literature on the prognostic value of SRI in patients with cancer is scarce. The SRI overexpression was reported to be closely related to the poor clinical outcomes and the complete remission rate in acute myeloid leukemia (45). Our Kaplan–Meier survival and the Cox regression analyses first demonstrated that SRI had predictive value on the survival outcomes in BLCA, BRCA, CESC, CHOL, HNSC, KIRP, KIRC, LGG, LIHC, OV, PAAD, PRAD, SKCM, STAD, THYM, THCA, UCEC, and UVM. In addition, the SRI expression was negatively associated with the disease stage in BLCA but positively correlated to the tumor stage in HNSC, LIHC, and MESO. Sorcin was previously reported to be positively related with the TNM stage in gastric cancer (46). Further, our study revealed that the high expression of SRI was significantly related to the with-tumor status in ESCA, GBM, KIRC, LIHC, PRAD, and UVM, suggesting SRI might have the potential for reflecting the tumor status.

Regarding the SRI genomic alterations in cancer, our data found that amplification was the most frequent mutation type in cancer. As reported in many studies, genomic amplification of the chromosomal region 7q21, such as ABCB1 and SRI occurred in the multidrug-resistant cancers (47–51). In treating with the chemotherapeutic drugs, amplification of the ABCB1-related amplicon region containing SRI was sufficient to drive tumor chemoresistance. Genomic amplification of SRI has long been recognized as an occasional event of such genomic co-amplification (52). However, accumulating evidence indicated that the pathways involved in the tumor malignant behaviors, such as TGF-β and JAK-STAT3 signaling were affected by the SRI gene amplification (35, 53). In addition, we found co-occurrence of ADAM22, DBF4, ABCB1, ABCB4, and RUNDC3B alterations was observed with the SRI alterations, which was consistent with the previous reports (48). The full-length sorcin could lead to a low level of paclitaxel resistance in ovarian cancer cells (54). In this study, our analysis showed that the amplification was associated with the high mRNA and protein level of SRI in ovarian cancer, which indicated the sorcin overexpression in ovarian cancer might be due in part to the SRI genomic amplification. A survival analysis further showed that the patients with cancer with the SRI genetic alterations had poorer OS than those with SRI unaltered group. The above findings suggested that the SRI genomic alterations were considered as a risk factor for prognosis in cancer.

The cancer stem cells (CSCs), a pool of specialized cancer cells, are at the root of tumor initiation and responsible for chemoresistance of malignant tumors (55). The occurrence of EMT endows cancer cells with the mesenchymal phenotypes and stem cell-like characteristics and thus confers invasiveness and chemoresistance (56). Sorcin silencing in the breast cancer cells decreases the pool of CD44+/CD24– and ALDH1 high CSCs *in vitro* (13). Sorcin was also reported to facilitate the migration, invasion, and EMT in breast cancer, ovarian cancer, and colorectal cancer (13, 14, 35). Sorcin overexpression is tightly associated with the increased local invasion and lymph node metastasis of gastric cancer (46). Mechanistically, sorcin silencing effectively decreased the expression of matrix metalloproteinases 2 and 9 (MMP2 and MMP9), and eventually suppressed gastric cancer metastasis (53). The previous studies have demonstrated that sorcin induced EMT-related phenotype by the regulation of PI3K/Akt/mTOR pathway and vascular endothelial growth factor (VEGF) (13, 14). Our analysis of on cancer stemness and EMT-related genes further supported the oncogenic and stemness-related role of SRI in cancer. MSI and TMB have recently captured widespread attention as promising predictive biomarkers for immunotherapy efficacy, especially in colorectal cancers and lung cancer (57, 58). Our results demonstrated that the SRI expression was significantly related to MSI in 12 cancer types and TMB in 13 cancer types. These results strongly implied that the SRI expression might affect the response to immune checkpoint therapy in patients with cancer, which will shed new light on the prognosis of immunotherapy. Further analysis revealed that the SRI expression was positively correlated with the MMR-related genes expression in most of the tumors. Thus, the patients with cancer with low expression of SRI, high MSI, and TMB may benefit from immunotherapy.

At present, the role of SRI in the tumor immune microenvironment remains a research gap worth investigation in further research. According to ESTIMATE algorithm, the correlation between the SRI expression and immune cell content might depend on the tumor types. TIMER database mining further found that the SRI expression was significantly correlated with the infiltration levels of various immune cells, particularly in ACC, ESCA, LIHC, LUSC, and PRAD. In addition, the correlation analysis demonstrated that the immune checkpoint genes were positively correlated with the SRI expression in most tumor types, suggesting SRI might be involved in immune escape. Further *in vitro* and *in vivo* studies exploring the relationship between the SRI expression and immune infiltrations are warranted. Currently, the SRI roles in multi-drug resistance have become increasingly appreciated. The previous studies reported sorcin knockdown resulted in the increased cisplatin, paclitaxel, doxorubicin, fluorouracil, and vincristine sensitivity in the cancer cells (21, 54, 59–62), which was also consistent with our drug sensitivity of SRI. Nevertheless, more experimental validation needs to be further studied to evaluate the influence of SRI on new drugs in clinical trials. Our findings may be useful in prioritizing further research on drug screening. Furthermore, our GSEA analyses suggested that SRI was closely associated with the metabolism-related pathways, transcription activator activity, immune-related pathways, and cell cycle G1/S transition. Relevant literature has reported the SRI affected glucose metabolism (63), STAT3 transcriptional activity (53), cell cycle progression in mitosis (64), and immuno-inflammatory responses (65). The results of GSEA further indicated that SRI was involved in immune-related pathways in certain types of tumor, which was consistent with our previous immune-related analysis on SRI. Our findings revealed that a signaling pathway was significantly enriched in various types of tumors. We, therefore, considered that the signaling pathways mediated by SRI were not specific for different cancers.

The pre-clinical studies have found that the natural compounds, such as dihydromyricetin and triptolide specifically reversed drug resistance through the downregulation of SRI expression *in vitro*, which indicates the clinical transfer value of SRI as a good candidate to prevent chemoresistance (22, 44). The significance of our work is that the multifaceted functions of SRI were unveiled in cancer, which not only further verified the previous findings but also advanced our understanding of the role mediated by SRI in the tumor immune microenvironment. Since our study was a comprehensive bioinformatics analysis and relied on multiple databases, several limitations are inevitable. First, the results are not experimental, and thus future experimental validations are required. Second, the majority of our analyses were focused on the mRNA expression of SRI. It is worth mentioning that the analyses based on the protein levels of SRI would make the results more convincing. Third, this work solely presented the correlation analysis, and the molecular mechanisms of SRI in tumor stemness and immune infiltration require further investigation in the future. To sum up, our pan-cancer analyses systematically probe the characteristics of SRI in multiple aspects, such as expression pattern, survival prognosis, genetic mutation, stemness, TMB, MSI, tumor immune microenvironment, and drug resistance. SRI might be a potential target for cancer therapy since it displayed abnormal high expression in multiple cancers and predicted worse prognosis in patients with cancer. Frequent amplification of SRI genomic was observed and the SRI expression correlated with amplification. Moreover, the aberrant SRI expression was related to the stemness score, EMT-related genes, MSI, TMB, and tumor immune microenvironment across various types of cancer. SRI was able to predict the sensitivity to chemotherapeutic agents as a novel resistance gene. The present study may provide insights for illustrating the role of SRI in tumorigenesis and drug resistance. At the same time, our work also points to several directions for future prospective studies focusing on SRI in cancer.

## Data Availability Statement

The original contributions presented in the study are included in the article/Supplementary Material, further inquiries can be directed to the corresponding author/s.

## Author Contributions

JZ performed most of the analyses and wrote the manuscript. XH and JZ conceived the idea and led the project. JC, BS, LL, JD, QS, and QZ performed some of the analyses and edited the manuscript. All authors contributed to the article and approved the submitted version.

## Funding

This work was supported by grants from the Key Science and Technology Program of Anhui province, China (1401042007).

## Conflict of Interest

The authors declare that the research was conducted in the absence of any commercial or financial relationships that could be construed as a potential conflict of interest.

## Publisher's Note

All claims expressed in this article are solely those of the authors and do not necessarily represent those of their affiliated organizations, or those of the publisher, the editors and the reviewers. Any product that may be evaluated in this article, or claim that may be made by its manufacturer, is not guaranteed or endorsed by the publisher.
