# Supplementary figures and images for: Clinical Significance and Prognostic Value of Human Soluble Resistance-Related Calcium-Binding Protein: A Pan-Cancer Analysis
Source: Front Med (Lausanne). 2021 Nov 16;8:752619. doi: 10.3389/fmed.2021.752619 (PMC8635117; doi:10.3389/fmed.2021.752619)

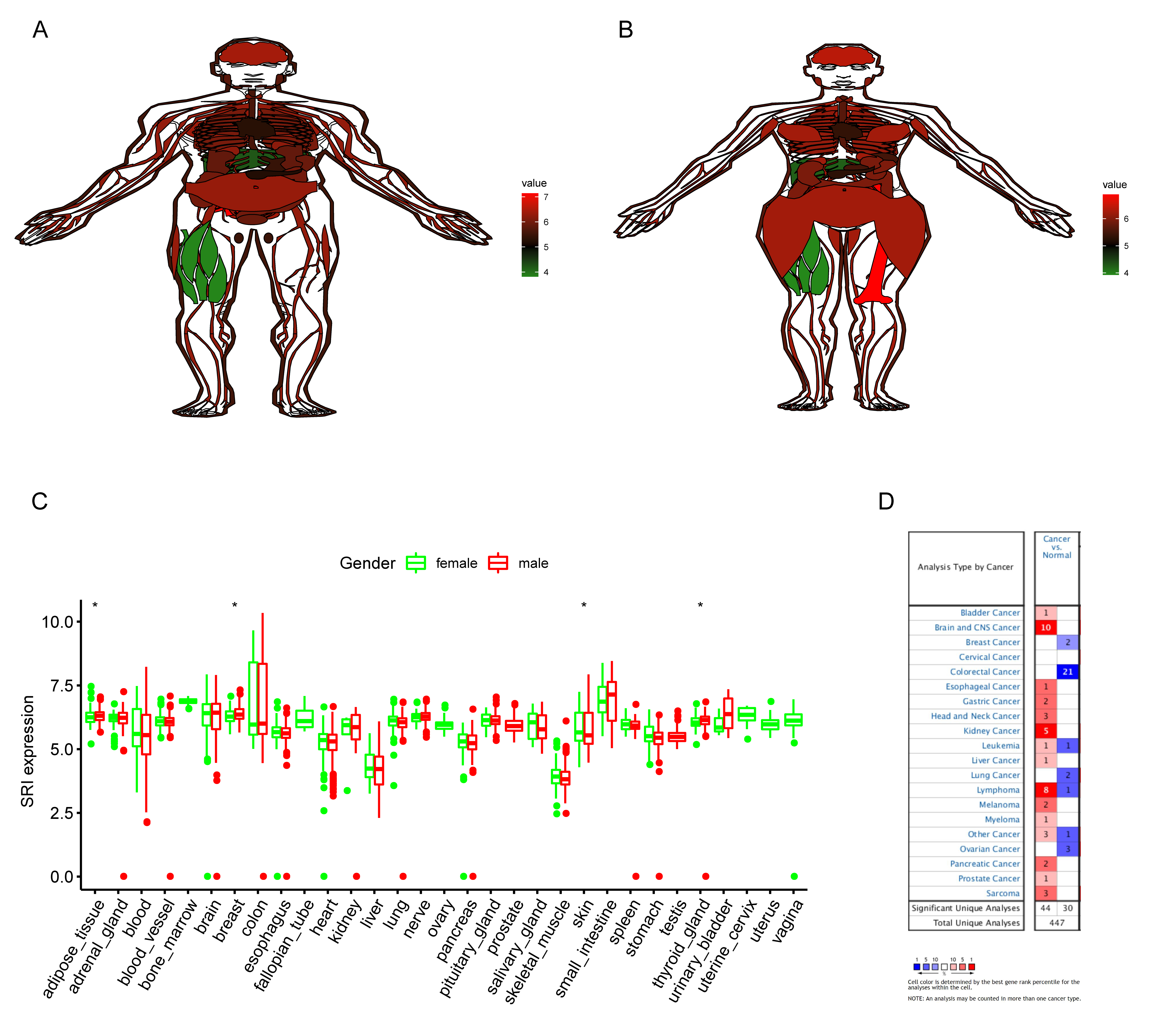

Supplement: Supplementary file 1 [file Image_1.tif]

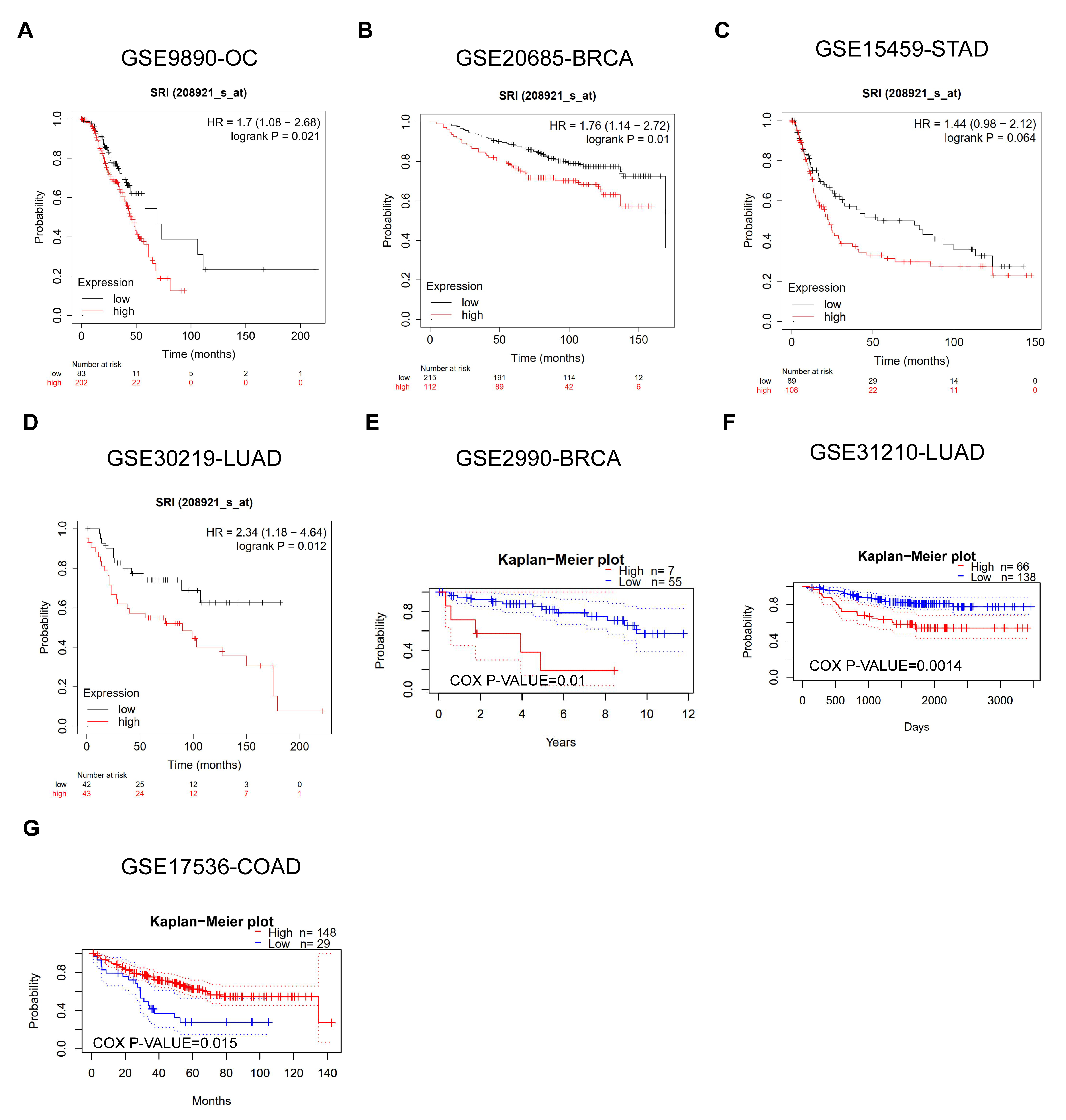

Supplement: Supplementary file 2 [file Image_2.tif]

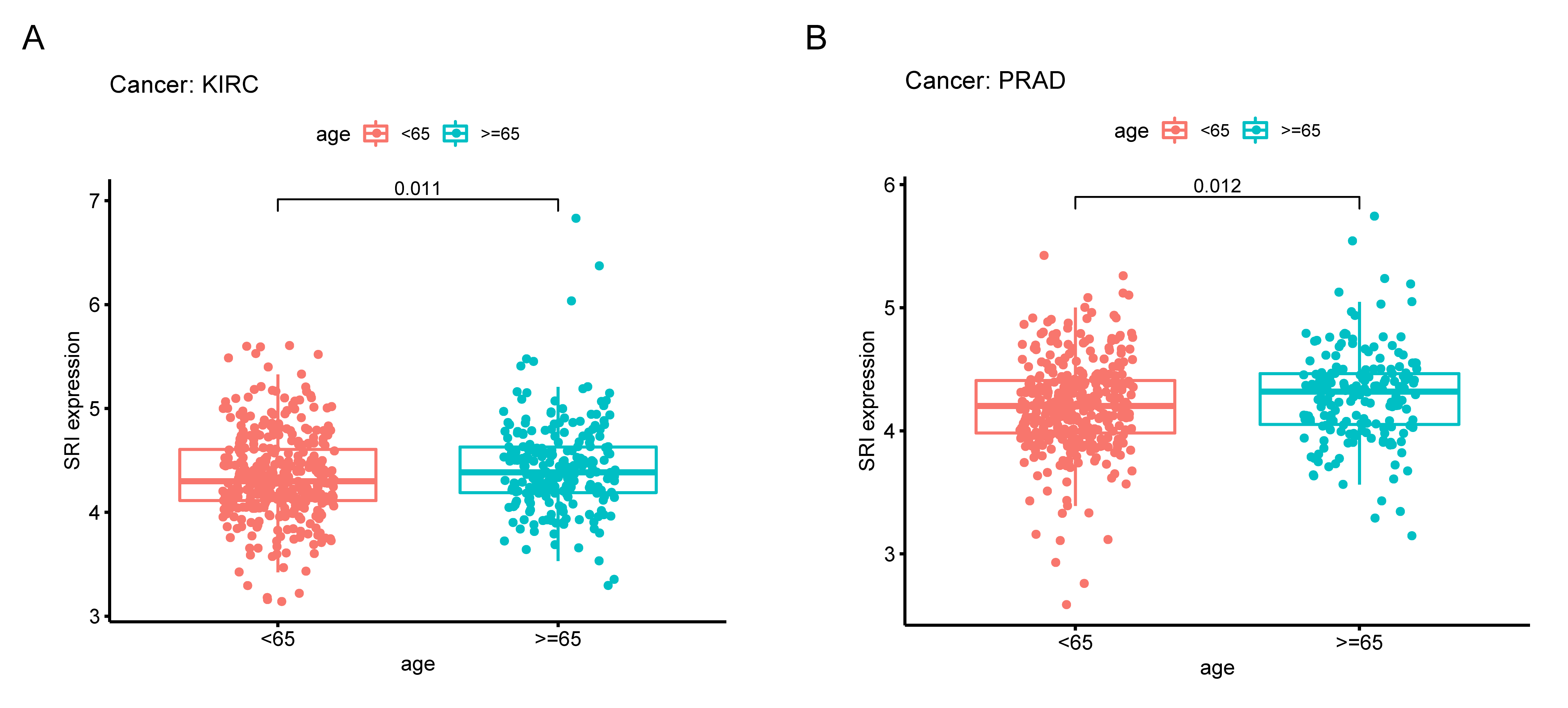

Supplement: Supplementary file 3 [file Image_3.tif]

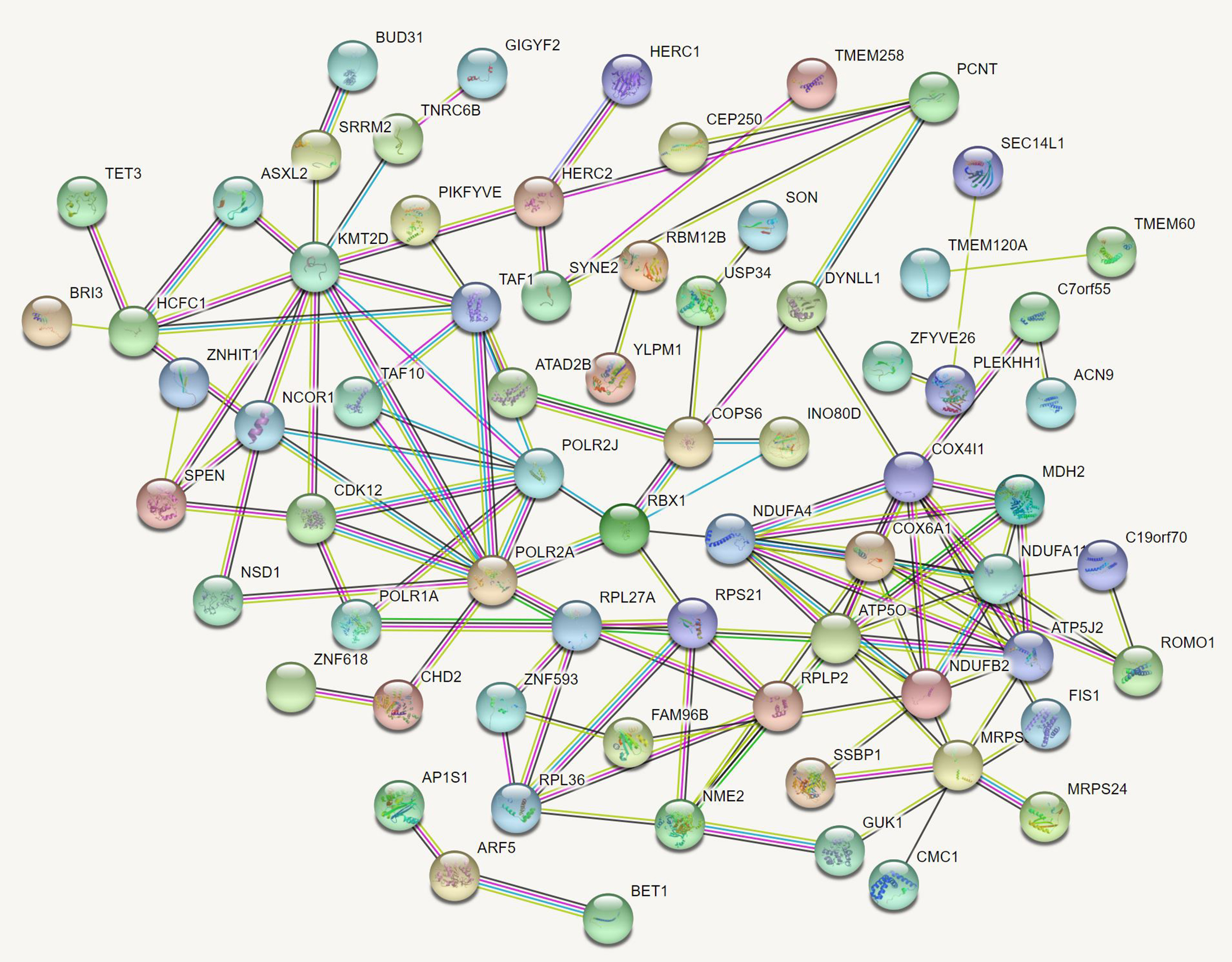

Supplement: Supplementary file 4 [file Image_4.jpg]

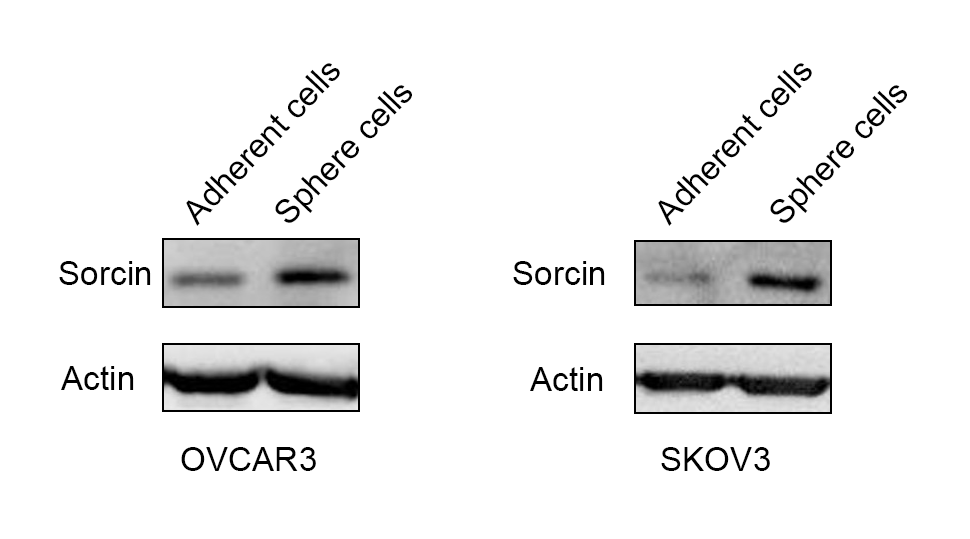

Supplement: Supplementary file 5 [file Image_5.tif]

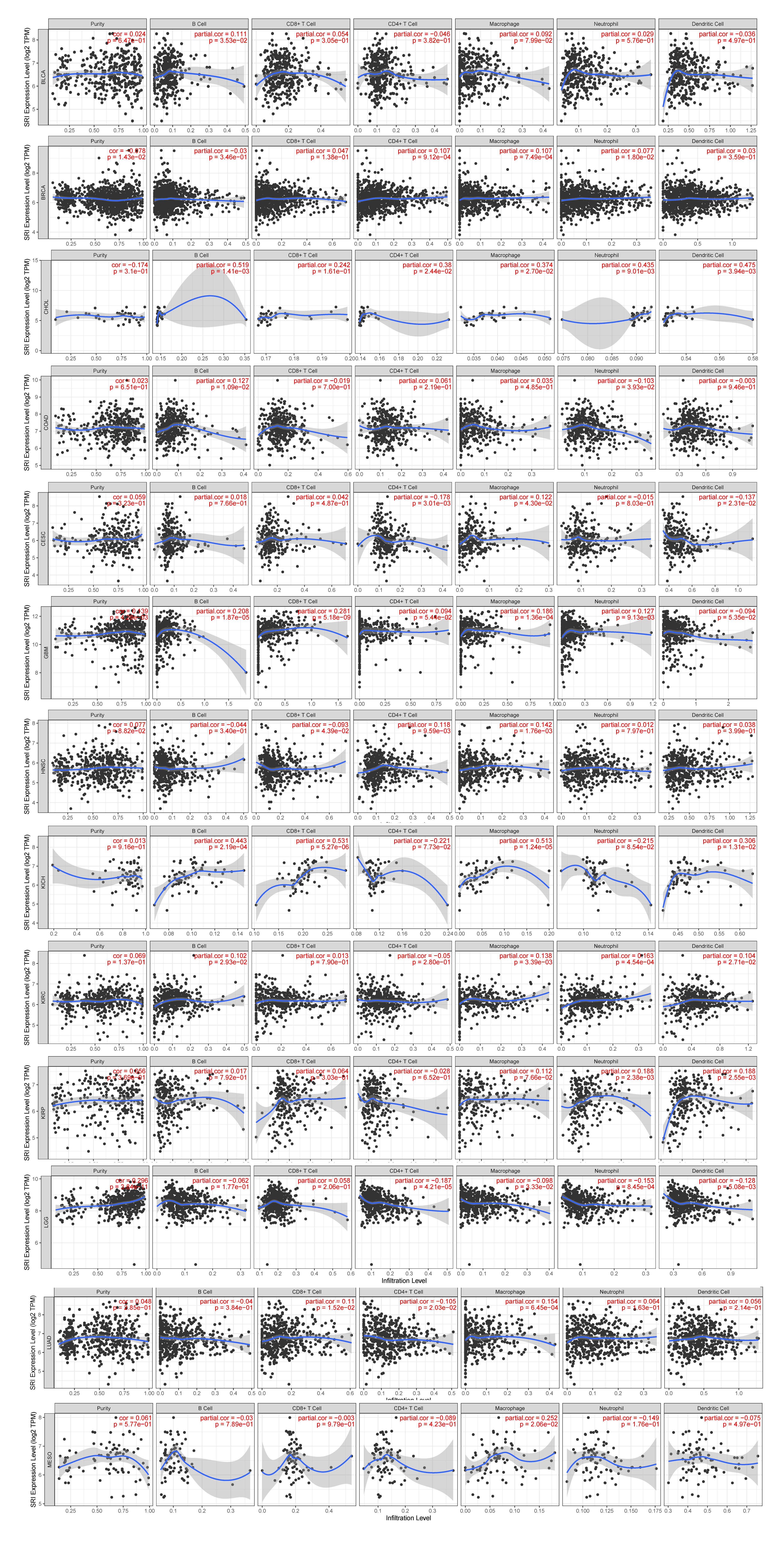

Supplement: Supplementary file 6 [file Image_6.tif]

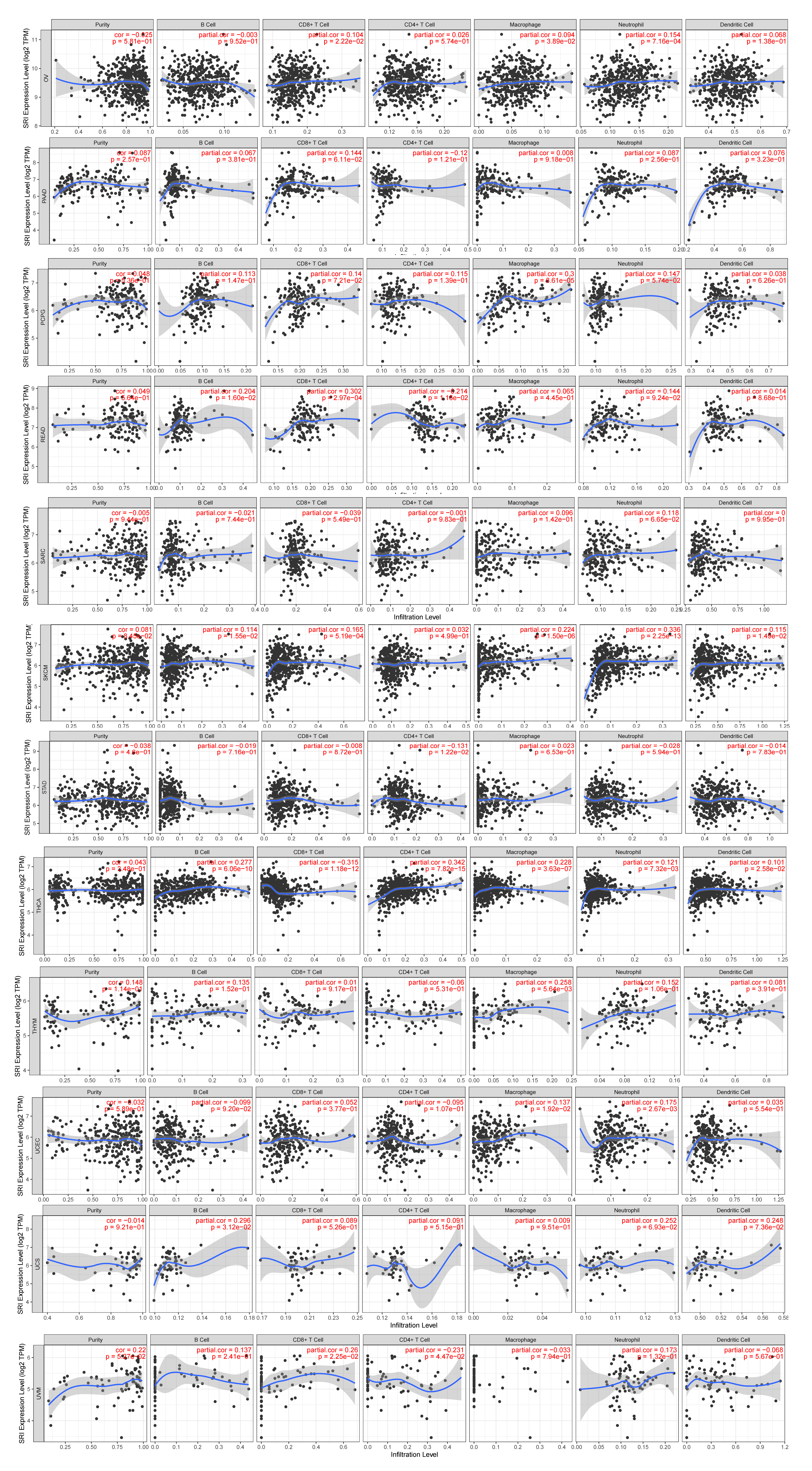

Supplement: Supplementary file 7 [file Image_7.tif]

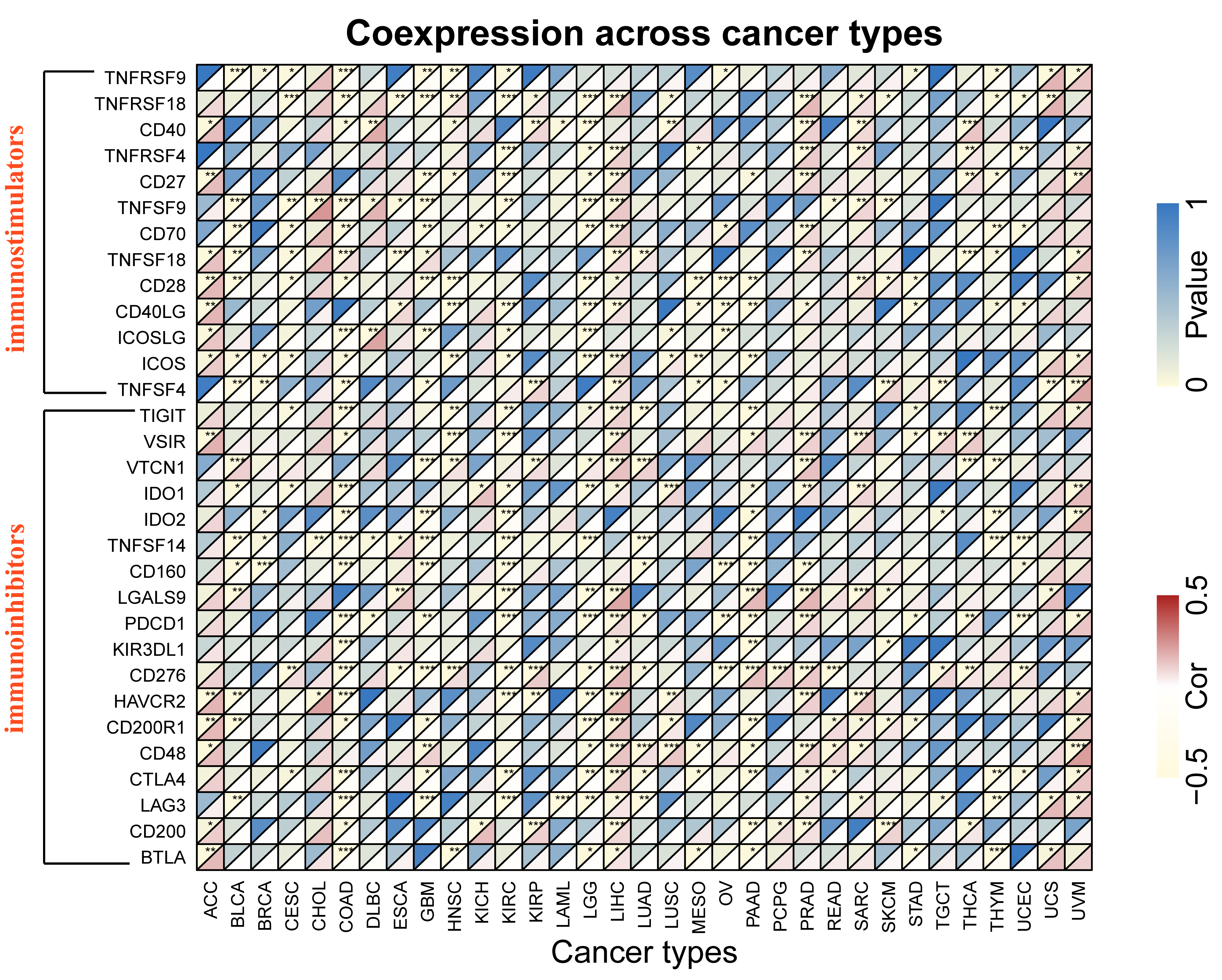

Supplement: Supplementary file 8 [file Image_8.tif]

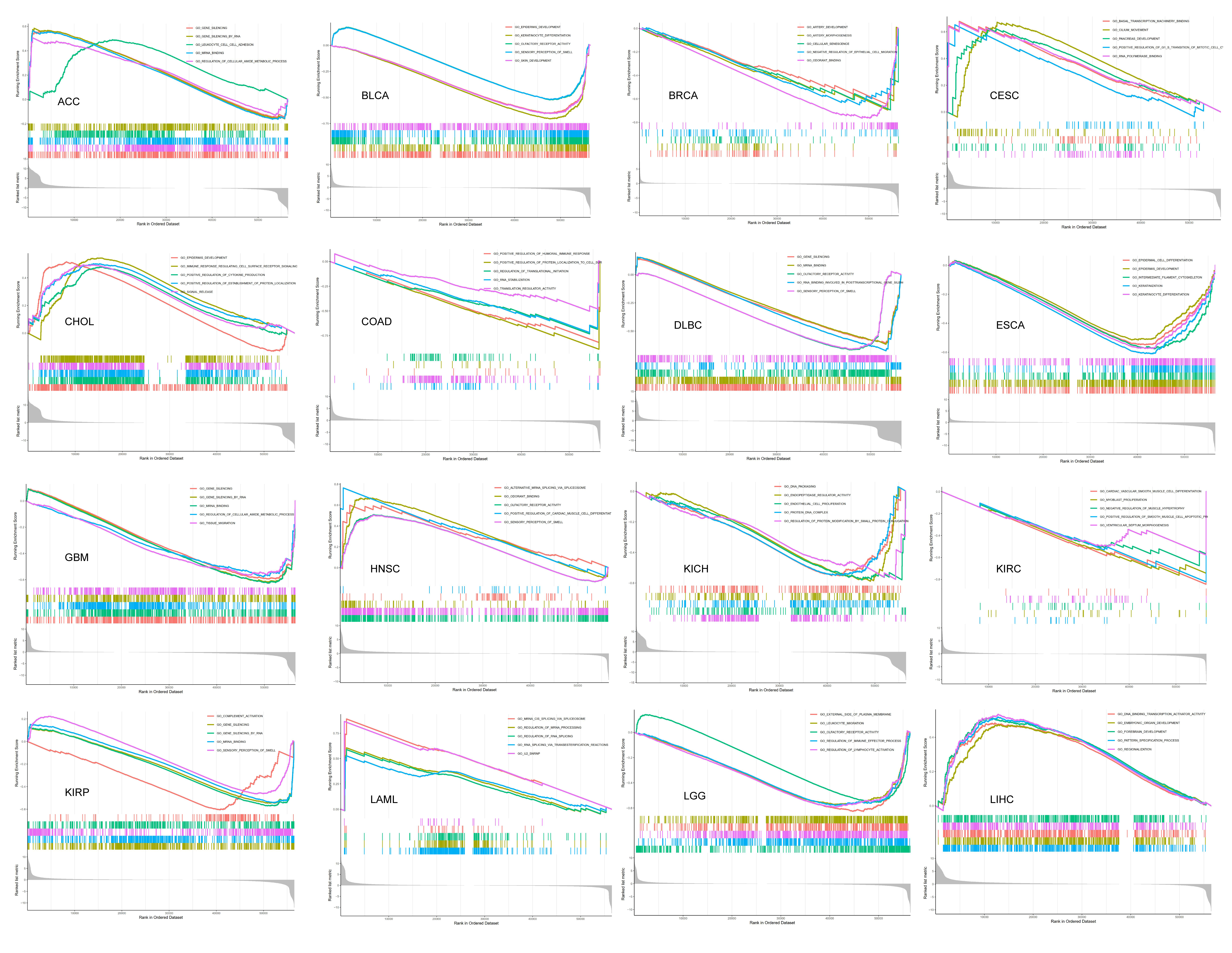

Supplement: Supplementary file 9 [file Image_9.tif]

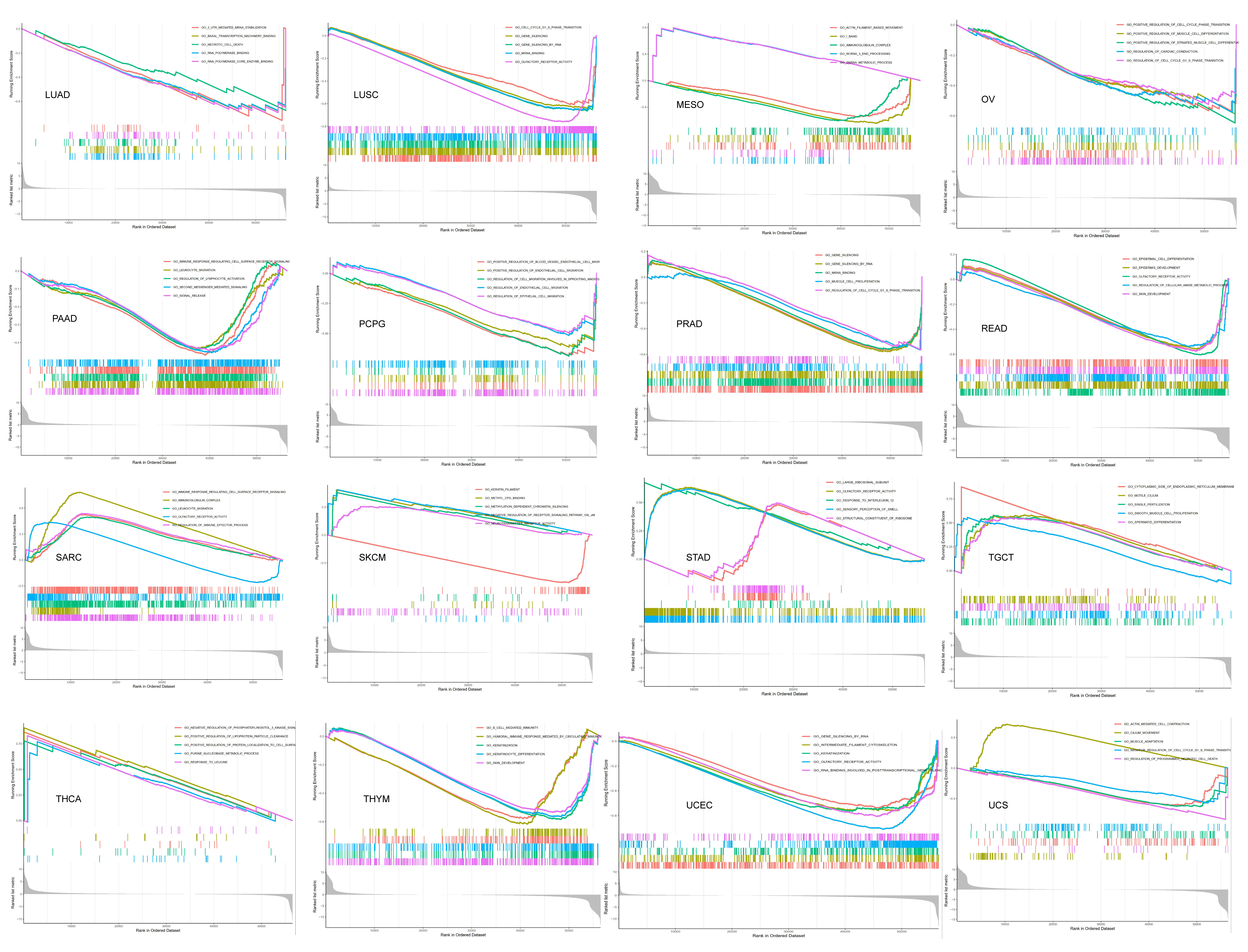

Supplement: Supplementary file 10 [file Image_10.tif]
